# Age patterns of under-5 mortality in sub-Saharan Africa during 1990–2018: A comparison of estimates from demographic surveillance with full birth histories and the historic record

**DOI:** 10.4054/demres.2021.44.18

**Published:** 2021-03-05

**Authors:** Hallie Eilerts, Julio Romero Prieto, Jeffrey W. Eaton, Georges Reniers

**Affiliations:** 1 Department of Population Health, London School of Hygiene and Tropical Medicine, London, UK.; 2 MRC Centre for Global Infectious Disease Analysis, School of Public Health, Imperial College London, London, UK.

## Abstract

**BACKGROUND:**

Estimates of under-5 mortality (U5M) for sub-Saharan African populations often rely heavily on full birth histories (FBHs) collected in surveys and model age patterns of mortality calibrated against vital statistics from other populations. Health and Demographic Surveillance Systems (HDSSs) are alternate sources of population-based data in much of sub-Saharan Africa, which are less formally utilized in estimation.

**OBJECTIVE:**

In this study we compare the age pattern of U5M in different African data sources (HDSSs, Demographic and Health Surveys (DHS), and Multiple Indicator Cluster Surveys (MICS)), and contrast these with the historical record as summarized in the Human Mortality Database and model age patterns.

**METHODS:**

We examined the relative levels of neonatal, postneonatal, infant, and child mortality across data sources. We directly compared estimates for DHS and MICS subnational regions with HDSS, and used linear regression to identify data and contextual attributes that correlated with the disparity between estimates.

**RESULTS:**

HDSS and FBH data suggests that African populations have higher levels of child mortality and lower infant mortality than the historic record. This age pattern is most explicit for Western African populations, but also characterizes data for other subregions. The comparison between HDSS and FBH data suggests that FBH estimates of child mortality are biased downward. The comparison is less conclusive for neonatal and infant mortality.

**CONTRIBUTION:**

This study questions the practice of using model age patterns derived from largely high-income settings for inferring or correcting U5M estimates for African populations. It also highlights the considerable uncertainty around the consistency of HDSS and FBH estimates of U5M.

## 1. Introduction

Ending preventable deaths of newborns and children under 5 years of age by 2030 is a central aim of the international community and is codified in the United Nations’ Sustainable Development Goal 3.2 (UN 2017). Accurate measurement of under-5 mortality (U5M) is essential to tracking and accelerating progress towards its reduction. Civil registration and vital statistics (CRVS) systems are the preferred sources for such data. However, in sub-Saharan Africa, the region with the highest U5M in the world, CRVS are often incomplete. For sub-Saharan Africa, estimates of U5M mostly rely on retrospective reporting of child survival in full birth histories (FBHs) collected in sample surveys and model age patterns of mortality (UN IGME 2018). However, biases in FBHs and uncertainty regarding the age pattern of U5M in sub-Saharan Africa give reason to regard such estimates with caution (Pullum and Becker 2014; Schoumaker 2014). The lack of reliable population-based data means there are few sources against which to validate these estimates. Health and Demographic Surveillance Systems (HDSSs) are designed to fill these data gaps.

HDSSs collect longitudinal data through closely spaced interviews of a geographically defined population. They are valuable sources of detailed, empirical data in low-resource settings across sub-Saharan Africa. However, the recording of vital events among individuals (including newborns) who join households between interview rounds can be incomplete and can deteriorate with less frequent rounds (Sankoh and Byass 2012).

In this contribution, we systematically compare the level and age pattern of U5M in HDSSs with FBH estimates from surveys and the historic record of high-quality CRVS data. A direct comparison between African data sources is used to investigate whether attributes of data collection, context, or quality can explain deviations from traditional age patterns of U5M. We use this approach to investigate the extent to which deviations from the historic record are driven by epidemiological factors or data quality issues. We find evidence that the age pattern of child mortality in sub-Saharan Africa deviates from the historical record assembled largely from CVRS in high-income settings. We also highlight the uncertainty in U5M estimation for sub-Saharan Africa, as well as the value to be gained from systematic comparison between HDSSs and other data sources.

## 2. Methods

### 2.1 Data

Our analysis makes use of data from HDSSs, FBHs in sample surveys, and high-quality CRVS.

HDSS data was obtained from the International Network for the Demographic Evaluation of Populations and their Health (INDEPTH Network) Data Repository.^3^ We included data from 30 studies in 13 African countries spanning 1990 to 2017.

The largest sources of FBHs for sub-Saharan Africa are the Demographic and Health Surveys (DHS) and UNICEF Multiple Indicator Cluster Surveys (MICS). We included data from 78 DHS conducted between 1995 and 2018 in 33 countries. For MICS, we included FBHs from 26 surveys conducted between 2010 and 2017 in 20 African countries. Data was procured from the DHS data repository^4^ and UNICEF MICS surveys webpage.^5^

Data from countries with high-quality CRVS was obtained from the Human Mortality Database (HMD).^6^ To improve the comparison with the sub-Saharan African data, we restricted data from the HMD to high-mortality contexts in which the level of U5M was between the 10th and 90th decile of DHS estimates. This included 99 life tables from 24 mostly European countries. Life tables which did not cover national populations were excluded (e.g., England and Wales Civilian Population, France Civilian Population, New Zealand Maori, New Zealand Non-Maori). Life tables for the total populations were excluded in favor of life tables for sub-populations with much longer time series (e.g., East Germany and West Germany were used instead of Germany, Total Population). The life table for Taiwan was excluded due to documented under-registration of infant deaths (Chen et al. 1998).

We used a model to summarize observed regularities in the age pattern of U5M in populations with high-quality data. The model was based on a new database of CRVS data compiled from historical yearbooks and the United Nations Statistical Division, and estimated using Wilmoth’s approach of flexible patterns of mortality (Wilmoth et al. 2012). It is a two-dimensional model with entry parameters for the overall level of U5M and the shape of the age pattern of mortality. More detail on the model is available elsewhere (Guillot et al. 2020).

### 2.2 Analysis

The analysis was conducted in three parts. First, we examined the age pattern of U5M in each data source. This consisted of a comparison of the relative levels of mortality for standard age breakdowns within the under-5 range. Next, we compared the absolute levels of mortality in HDSSs with subnational region estimates from DHS and MICS. In the final part of the analysis we used ordinary least squares (OLS) regression to identify fieldwork practices, contextual attributes, and indicators of data quality that correlated with the relative disparity between HDSS and DHS estimates.

#### 2.2.1 Age pattern of U5M

We compared the distribution of mortality under age 5 in each data source using the neonatal, postneonatal, infant, and child age groups. Neonatal mortality refers to the probability of dying in the first 28 days of life, and is denoted as *q*(28*d*). Postneonatal mortality is the probability of dying before age 1, conditional on surviving the first month of life (*q*(28*d*, 12*m*)). Infant mortality is the probability of dying between birth and exact age 1 (*q*(12*m*)), and child mortality is the probability that a child aged 1 year will die prior to reaching age 5 (*q*(12*m*, 5y)). Under-5 mortality is the probability of dying between birth and exact age 5 (q(5y)).

In order to calculate mortality estimates from HDSS data, we used individual-level records with exact dates of births, deaths, migrations, and censoring to assign events and exposure time to 5-year periods extending backwards from the most recent year of available data. Within each period, events and exposure time were further disaggregated into age groups consisting of segments of 0, 1–2, 3–4, 5–11, 12–23, 24–35, 36–47, and 48–59 completed months. We performed a standard demographic calculation of observed deaths over person-years to calculate the mortality rate for each age group. Piece-wise constant rates were aggregated over age groups to produce the cumulative hazard of mortality between the ages 0 and 28 days, 28 days and 1 year, 0 and 1 year, 1 and 5 years, and 0 to 5 years. We converted mortality rates into probabilities of dying *q*(28*d*), *q*(28*d*, 12*m*), *q*(12*m*), *q*(12*m*, 5y), and *q*(5y).

It would be preferable to begin exposure time when births or in-migrations were first observed by the HDSS (the observation date), rather than the date of the events themselves. This is due to under-ascertainment of left-censored vital events, where the vital events of individuals that do not survive to the first HDSS round are less likely to be reported. However, dates of observation have not been universally recorded across sites and time. As a sensitivity analysis, we recalculated estimates of mortality with exposure starting at the observation date when such data was available, and compared to the standard estimates.

Estimates of mortality from DHS and MICS surveys were calculated using the retrospectively reported information of FBHs. We used FBHs to assign births, deaths, and exposure time to age groups in the 0–4 and 5–9 years prior to each survey. We calculated national-level estimates of *q*(28*d*), *q*(28*d*, 12*m*), *q*(12*m*), *q*(12*m*, 5y), and *q*(5y) for each 5-year period following the same methods as described for HDSS data. For MICS surveys that were not conducted at the national-level, estimates were calculated for the entire sub-population included in the survey. For the HMD, estimates of national-level *q*(12*m*), q(12*m*, 5y), and *q*(5y) were available in 5-year period abridged life tables.

The age pattern of U5M in each source was evaluated in a descriptive manner. We limited estimates for African contexts to periods between 2000 and 2018. This was done to avoid comparing the age pattern of mortality across vastly different levels of mortality. The overall level of mortality plays an important role in determining the age pattern, though the exact nature of this relationship has been difficult to establish for countries in sub-Saharan Africa (Blacker, Hill, and Timaeus 1985; Guillot et al. 2012). The exclusion of data prior to 2000 was also justified by the lack of MICS FBHs prior to this time. We examined the distribution of mortality under age 5 by plotting estimates of infant mortality against child mortality. For mortality under age 1, the distribution was further stratified by a scatter plot of neonatal and postneonatal mortality. We also plotted the ratio of infant-to-child mortality against under-5 mortality, and the of postneonatal-to-neonatal mortality against infant mortality.

We included a model prediction on the scatter plots displaying the distribution of mortality under 1 and 5 in each data source. The model is a log-quadratic representation of the cumulative probability of dying as a function of the level of U5M and a continuous parameter modifying the shape of the pattern of mortality (Guillot et al. 2020). We calculated the model predictions for postneonatal mortality using predefined values of neonatal mortality (that covered the observed range of DHS estimates), and five different shape parameters. With these inputs, we used the model to solve the level of U5M and indirectly estimate infant mortality. We then subtracted the predefined values of neonatal mortality from the model’s infant mortality estimates to produce estimates of postneonatal mortality. The shape parameters were selected to produce estimates of the model’s central tendency and inner and outer bounds. The bounds represent values of postneonatal mortality corresponding to different patterns of mortality, with the outer bounds representing particularly extreme age patterns. We used the same procedure to indirectly estimate child mortality using predefined values of infant mortality.

We summarized the ratios of child-to-infant and postneonatal-to-neonatal mortality in HDSSs, FBHs, and the HMD with boxplots. We further disaggregated the ratios by African subregion (Southern, Eastern, Central, Western) using the United Nations geoscheme, and compared them using median, interquartile range (IQR), and non-parametric tests of differences in median (with the DHS median as the reference). We further stratified the Western region to analyze whether the ratios differed for Coastal and Sahelian countries.

#### 2.2.2 HDSS and FBH differences

We then conducted a direct comparison of HDSS and FBH mortality estimates. We recalculated mortality estimates from DHS and MICS at the subnational region level for the 0–4 and 5–9 years prior to all surveys administered between 1995 and 2018. HDSS were mapped to the DHS and MICS subnational regions using GPS coordinates for each site’s approximate location, taking into account changes in survey region names and boundaries over time. We calculated HDSS mortality estimates for the same five-year periods as the FBH estimates. We calculated the relative percentage differences between HDSS and FBH estimates by subtracting the FBH values from the HDSS, dividing by the average of the two values, and multiplying the quotient by 100. We examined the distribution of differences between sources in boxplots.

#### 2.2.3 Factors associated with HDSS–DHS differences

Finally, we used OLS regression to identify predictors of the size of the discrepancy between HDSS and DHS subnational region estimates. The relative percentage differences served as the response variable in linear regression models with covariates related to HDSS and DHS fieldwork practices, contextual attributes, and indicators of data quality. We used bivariate models to investigate associations with relative differences in neonatal, postneonatal, infant, and child mortality. We added covariates which were strongly associated with the response variables to multivariable linear regression models. The comparison between HDSS and MICS subnational region estimates was not included in this part of the analysis due to having too few overlapping observations for analysis.

As covariates related to HDSS fieldwork practices, we included the interval between HDSS rounds (in months), and whether the site conducts pregnancy reporting. Pregnancy reporting refers to a system of collecting information on pregnancy status for female residents of reproductive age during each regular data collection round, and using this information to follow-up on pregnancy outcomes in subsequent rounds. We included HDSS digit preference for day of death reporting as a data quality covariate. We measured digit preference during the 5-year periods of observation using the Index of Dissimilarity, which compares the observed and expected distributions of day of death reporting for calendar days 1–31. The expected distribution of reports was taken from the frequency of each day throughout the year (12/365 for days 1–28, 11/365 for days 29 and 30, and 7/365 for day 31). We calculated the index by summing the absolute deviation of the observed distribution of reports from the expected distribution and dividing by two. We also included the log value of the average annual births in the HDSSs as a contextual attribute of interest.

For DHS data attributes we included markers of data quality, adult mortality, and the ‘time prior to the survey’ (TIPS). We calculated the data quality covariate by combining indicators for age heaping, date of birth displacement, and date of birth incompleteness in DHS FBHs. These indicators were calculated for each survey at the subnational region level following methods described elsewhere (Pullum and Staveteig 2017). We calculated adult mortality as the probability of dying between ages 15 and 50 using data from DHS sibling survival modules. This covariate was used as a proxy variable for potential bias in U5M estimates from missing mothers in high-HIV settings. The TIPS covariate was a dummy variable indicating whether the mortality estimate was for the period 0–4 or 5–9 years prior to the survey. In DHS FBHs, health information is collected on children born during the period 0–4 years prior to the administration of survey, and DHS fieldworkers sometimes displace events outside of this window in order to reduce their workload (Pullum and Becker 2014; Schoumaker 2011). We used this covariate to investigate the effect of such transfers on mortality estimates.

We included malaria endemicity as a contextual attribute of interest. This was calculated as the plasmodium falciparum parasite rate in 2–10 year olds at the approximate GPS location of each HDSS using data from the Malaria Atlas Project’s (MAP) global database.^7^ We also included African subregion of the HDSS/DHS as a covariate.

All analyses were done in R version 3.6.1. We used the rdhs and demogsurv R packages to assist with data procurement and calculations (MRC Centre for Global Infectious Disease Analysis 2017; Watson, FitzJohn, and Eaton 2019).

## 3. Results

**Figure 1:**
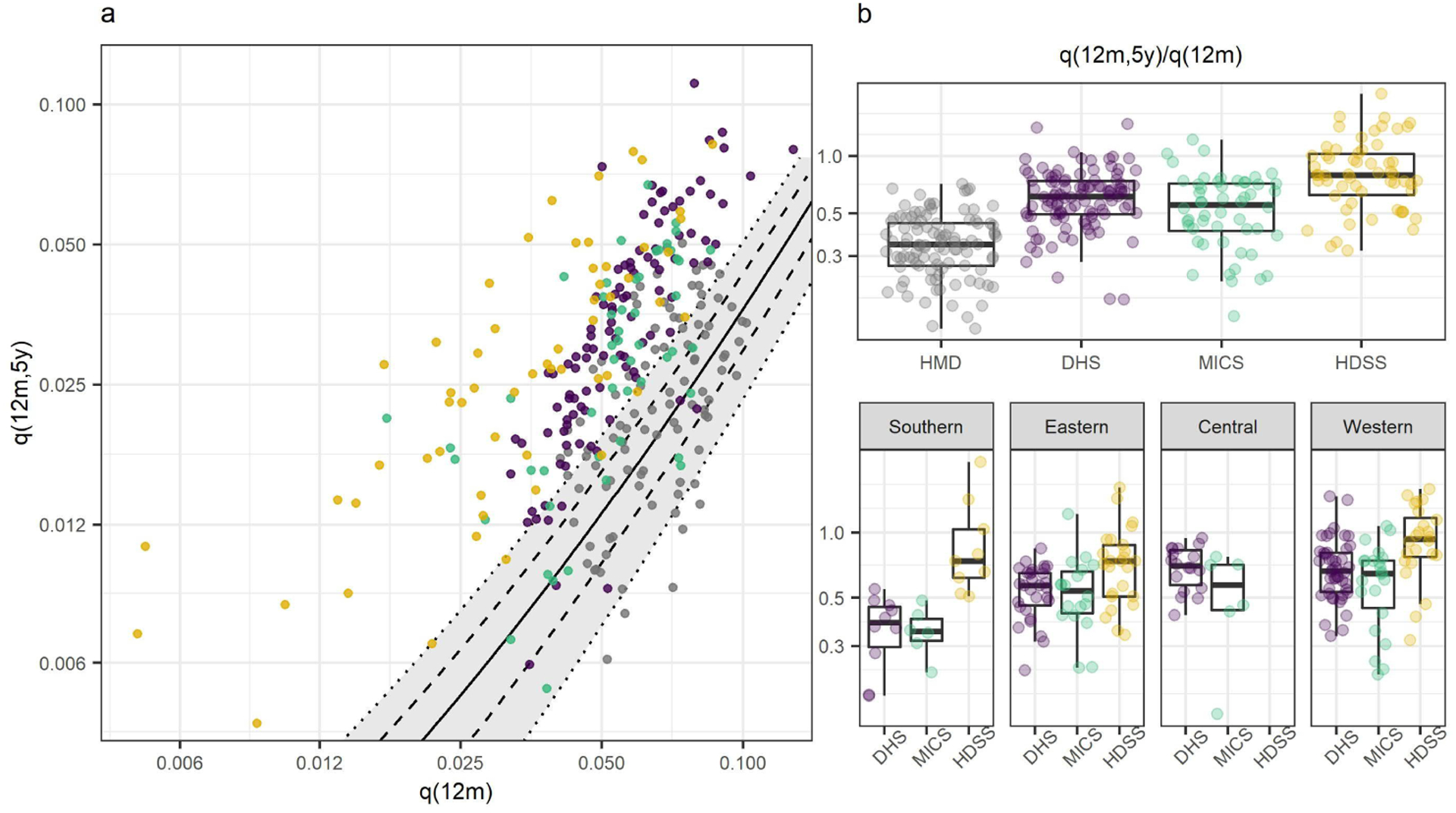
Age pattern of mortality under 5 *Note:* a: Scatter plot showing the relationship between infant and child mortality in HDSS, DHS, and MICS from sub-Saharan Africa, and high-mortality observations from the HMD. The black solid line is the model estimate of the relationship between the two indicators, derived from high-quality CRVS data. Dashed and dotted lines indicate model predictions under different age patterns of U5M. b: Box plots showing the ratio of child-to-infant mortality in each source, and disaggregated by African subregion.

**Figure A-1:**
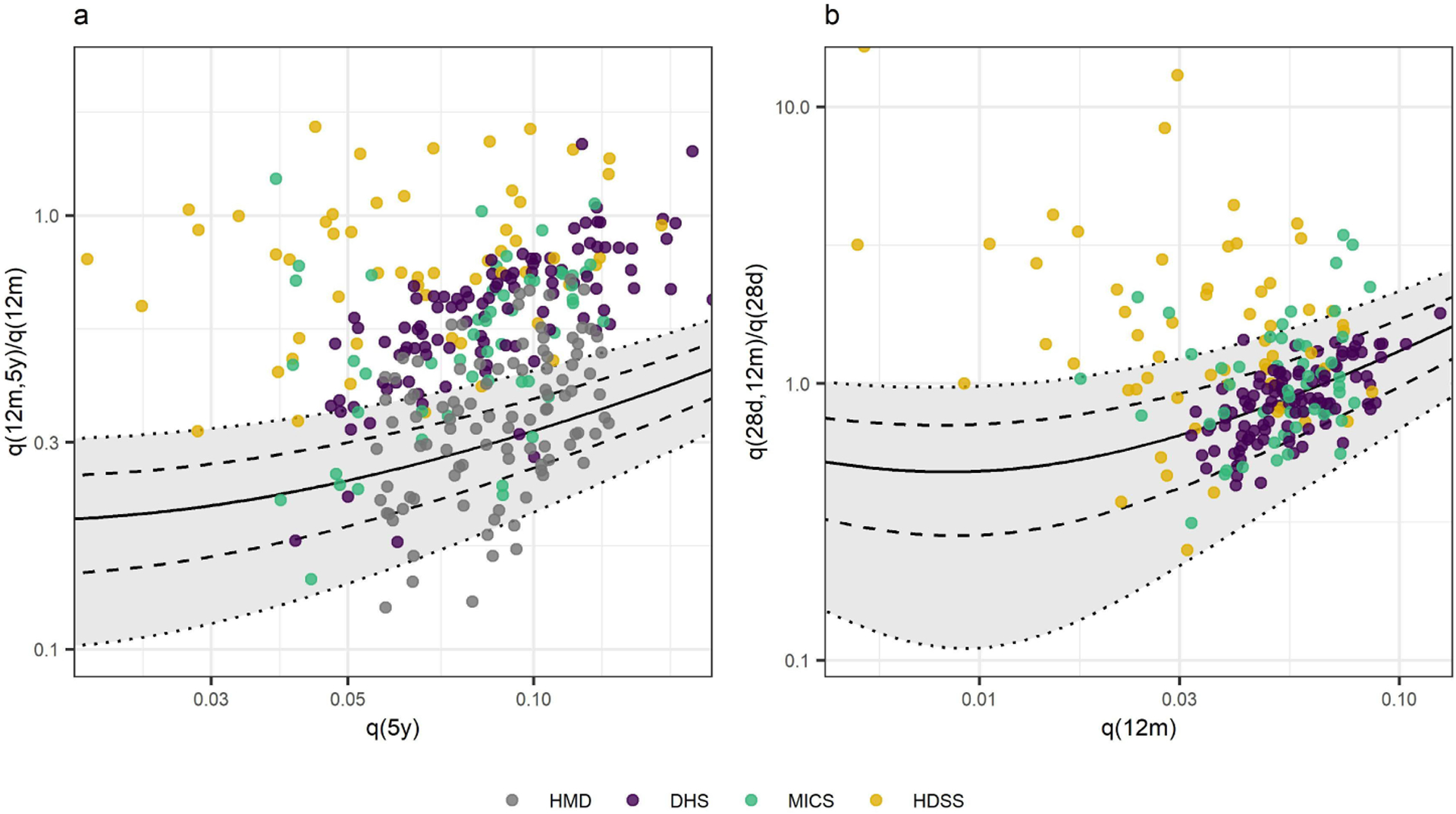
Age pattern of mortality against overall level of mortality *Note:* a: Scatter plot of the ratio of child-to-infant mortality against the overall level of mortality under 5. b: Scatter plot of the ratio of postneonatal-to-neonatal mortality against the overall level of mortality under 1. The black solid line is the model estimate of the relationship between the two indicators on each plot, derived from high-quality CRVS data. Dashed and dotted lines indicate model predictions under different age patterns of mortality.

The relationship between infant and child mortality in each data source is displayed in Figure 1. In Figure 1a, the relationship between infant (*q*(12*m*)) and child *q*(12*m*, 5y)) mortality in the HMD is similar to that which is portrayed with the model. However, the pattern is very different for African data sources. Data from HDSS, DHS, and MICS have higher levels of child mortality for given levels of infant mortality than the historic record and model predictions. This was consistent across different levels of under-5 mortality (panel A of Figure A-1).

**Figure A-2:**
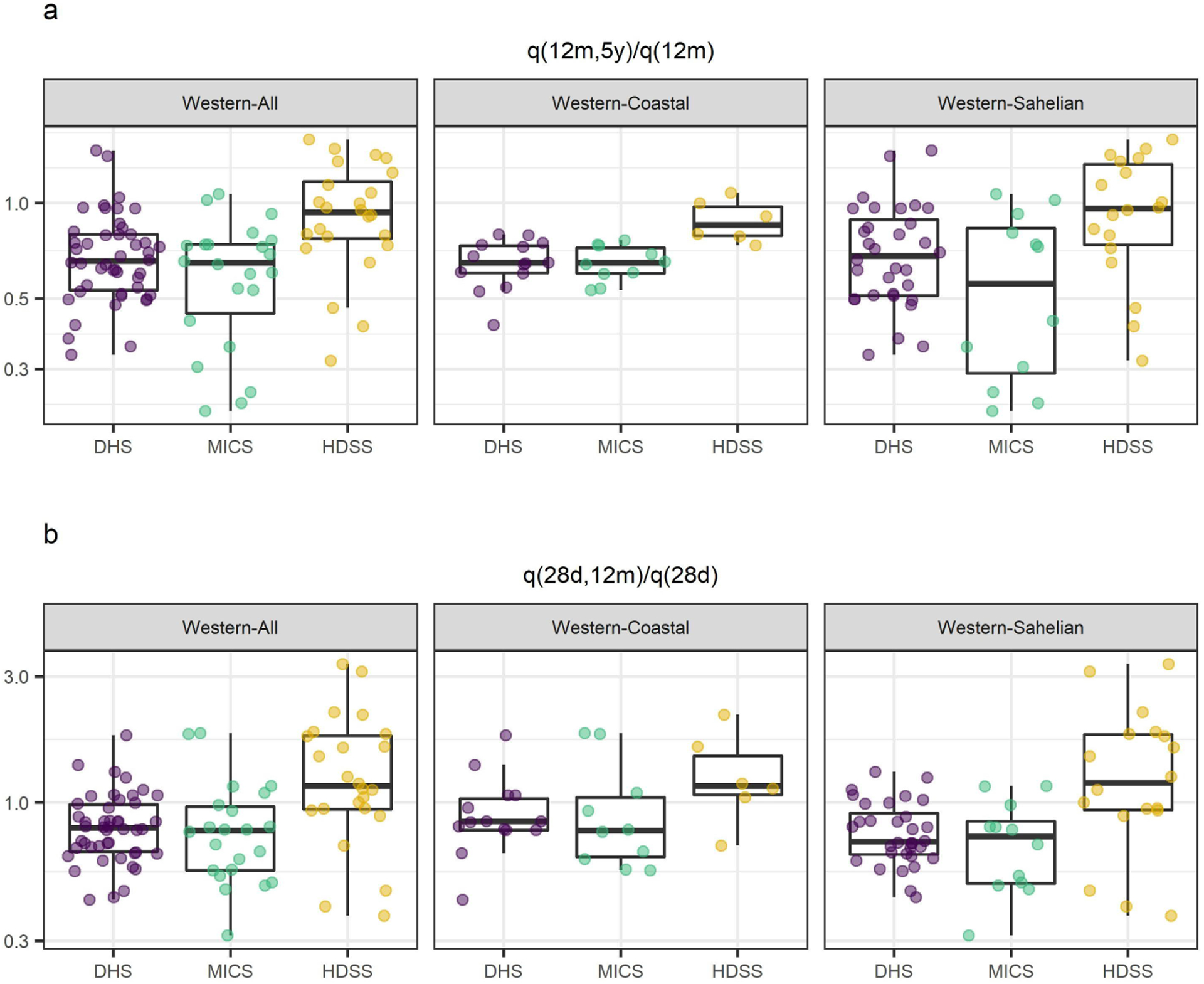
Mortality ratios for Coastal and Sahelian countries in West Africa *Note:* Box plots showing the ratio of child-to-infant mortality and postneonatal to neonatal mortality in each data source for all countries in the Western Region, and disaggregated by coastal and Sahelian countries.

Figure 1b displays the same data as panel A, but in the form of the ratio of child-to-infant mortality. The median ratios of child-to-infant mortality from African estimates were substantially higher than the HMD median of 0.34. The ratio was the highest in HDSS (0.79), followed by DHS (0.61) and MICS (0.55). When examined by African subregion, the HDSS ratio in Southern Africa was most different from the FBH data (p<0.01). The highest median ratios for HDSS and MICS were in Western Africa (0.93 and 0.65, respectively). A breakdown of the western region found that this was mostly driven by HDSSs in Sahelian countries rather than Coastal countries (panel A of Figure A-2). The highest median ratios for DHS were in the central and western regions (0.71 and 0.66, respectively). Full results for the ratios of child-to-infant mortality are shown in Table A-1.

**Figure 2:**
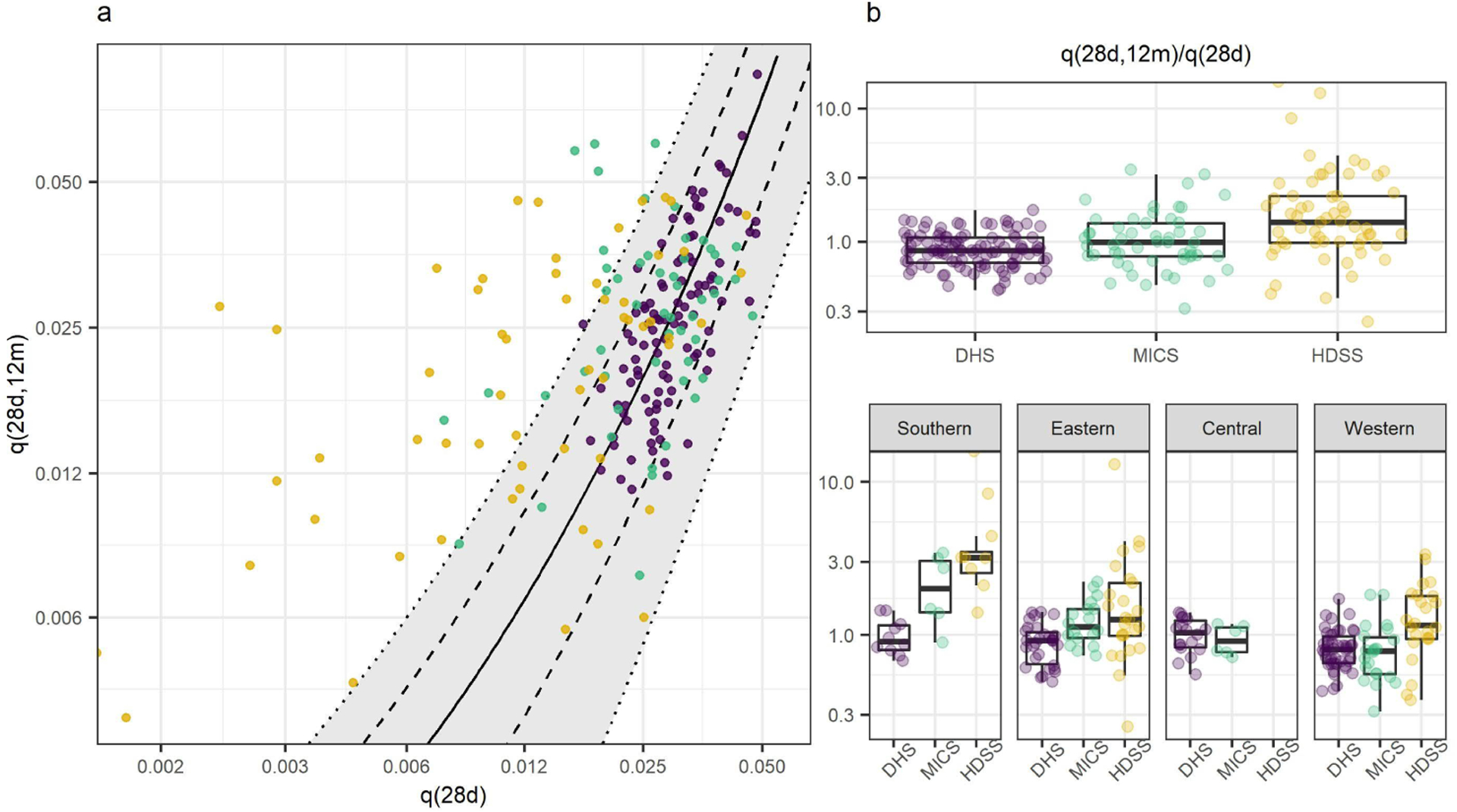
Age pattern of mortality under 1 *Note:* a: Scatter plot showing the relationship between neonatal and postneonatal mortality in HDSS, DHS, and MICS from sub-Saharan Africa. The black solid line is the model estimate of the relationship between the two indicators, derived from high-quality CRVS data. Dashed and dotted lines indicate model predictions under different age patterns of U5M. b: Box plots showing the ratio of postneonatal-to-neonatal mortality in each data source, and disaggregated by African subregion.

Figure 2 displays the relationship of neonatal (*q*(28*d*)) and postneonatal (*q*(28*d*, 12*m*)) mortality. In Figure 2a the levels of neonatal mortality in HDSSs were strikingly low compared to MICS, DHS, and modeled estimates. This resulted in HDSSs having mostly lower overall mortality in the infant period than other sources, but extremely high ratios of postneonatal-to-neonatal mortality (panel B of Figure A-1). The relationship between neonatal and postneonatal mortality in FBHs was consistent with modeled patterns from high quality CRVS, particularly for DHS.

Figure 2b displays the ratio of postneonatal-to-neonatal mortality in each data source, and disaggregated by African subregion. The HDSS data had the highest median ratio of 1.41, compared to the MICS median of 0.98 and DHS of 0.85. HDSS ratios were also generally higher when compared to FBHs from the same African subregions.

In the southern region the MICS median was double that observed in the DHS (2.10 compared to 0.91, p = 0.01). HDSSs in the southern region had the highest median ratio, with a level of postneonatal mortality 3.19 times that of neonatal mortality. In the western region more than 75% of HDSS ratios were higher than the DHS median (p < 0.01). For HDSSs and MICS there was not a clear difference in the ratio of postneonatal-to-neonatal mortality for Coastal and Sahelian West African countries (panel B of Figure A-2). For DHS the ratio was higher in Coastal countries (0.85 compared to 0.71). The full results for ratios of postneonatal-to-neonatal mortality are shown in Table A-2.

**Figure 3:**
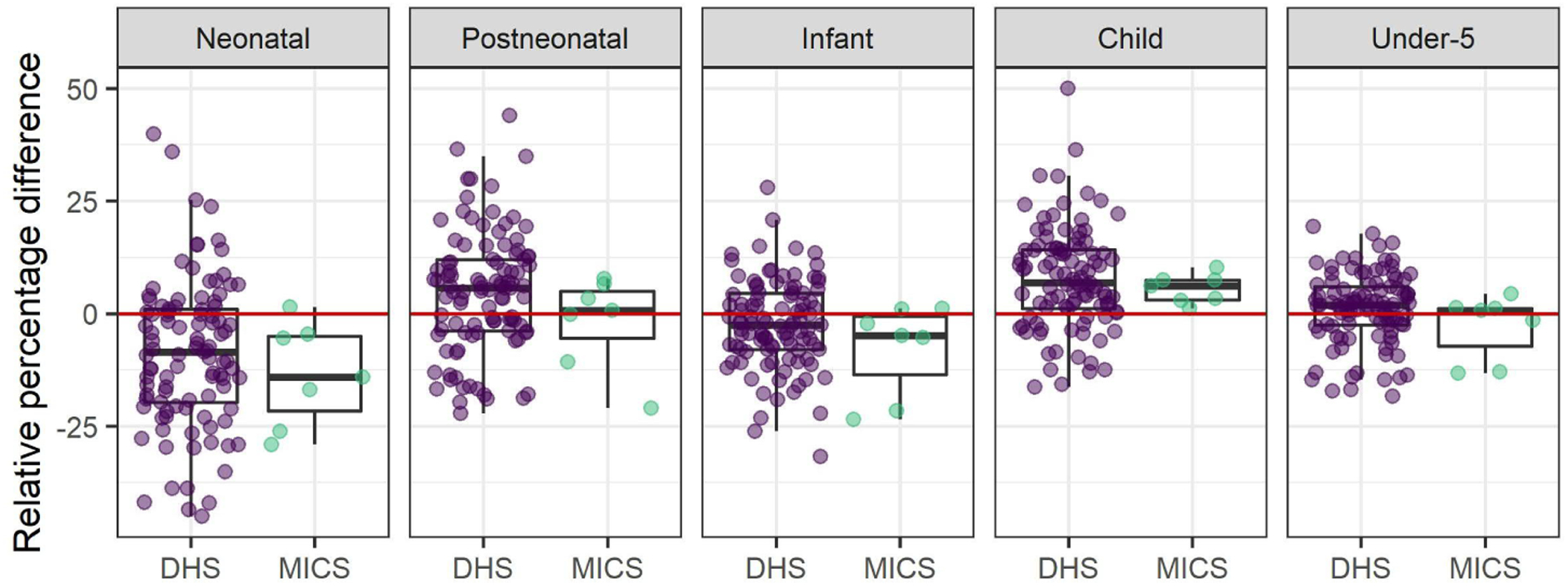
HDSS-FBH differences *Note:* Relative percentage differences between HDSS and DHS/MICS subnational region mortality estimates for overlapping 5-year periods.

Subnational regions from DHS and MICS were matched with HDSSs located within the regions to directly compare U5M estimates. There were 101 overlapping 5-year period estimates for HDSS and DHS, and 7 for HDSS and MICS. This included data from 24 HDSS sites, 31 DHS surveys, and 3 MICS surveys. Figure 3 shows the distribution of relative percentage differences between HDSS and FBH estimates of neonatal, postneonatal, infant, child, and under-5 mortality. Positive values indicate higher estimates from the HDSS, while negative values indicate higher estimates from DHS or MICS.

HDSS estimates of neonatal mortality were typically lower than DHS and MICS. The median HDSS value was 8.48% lower than the corresponding DHS subnational region estimate, and 14.10% lower than the MICS estimate. The reverse was the case for postneonatal mortality: the median HDSS values were 5.68% and 0.75% higher than DHS and MICS medians, respectively. When neonatal and postneonatal ages were combined for estimates of infant mortality, the HDSS median was 2.52% lower than the DHS, and 4.83% lower than MICS. For child mortality, the median HDSS estimate was 6.90% higher than the DHS and 6.14% higher than MICS. Altogether, HDSSs median U5M estimates were roughly 1.85% higher than DHS and 0.64% higher than MICS.

**Figure A-3:**
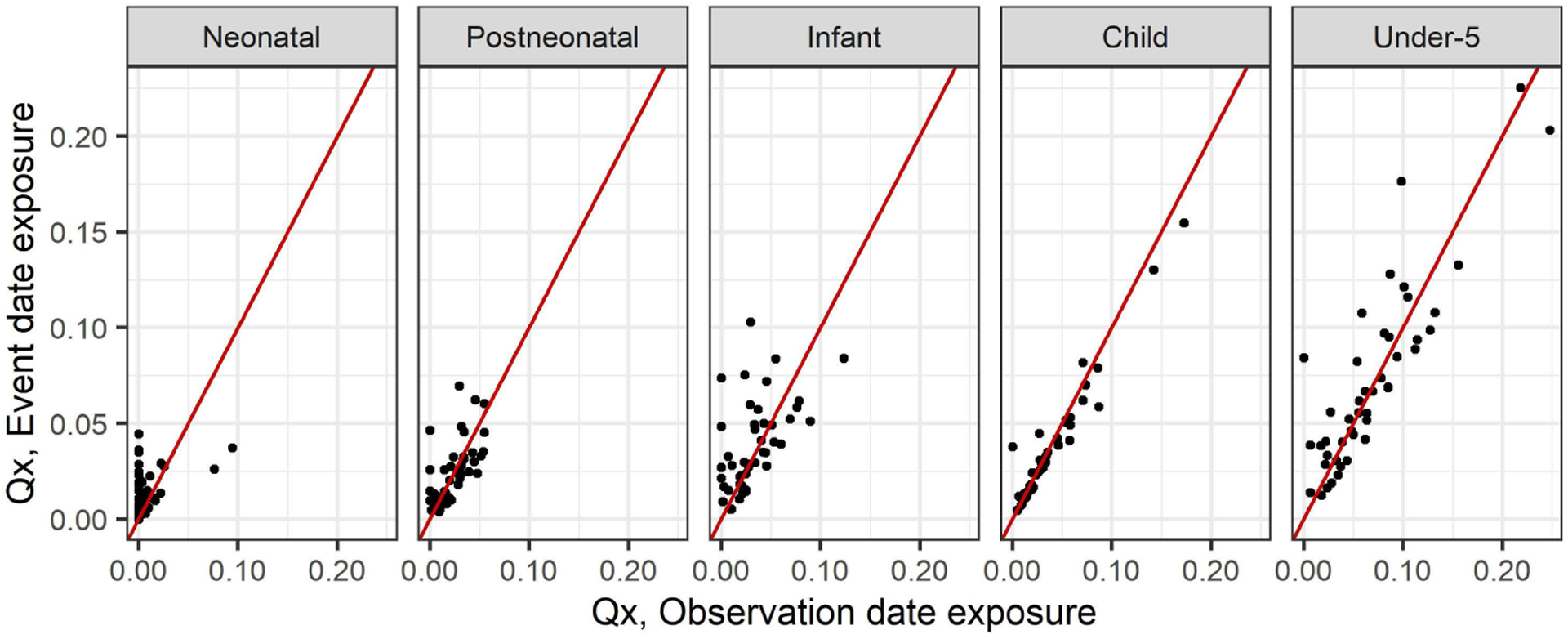
Comparison of HDSS mortality estimates with exposure beginning at event date versus observation date *Note:* This comparison uses a subset of the HDSS data from 23 sites. Mortality estimates with exposure beginning at observation date were calculated for 45 5-year periods where observation dates were being recorded consistently. These were compared to standard estimates where exposure time was calculated from the date of the birth/in-migration (event date). Neonatal mortality estimates are generally higher using event date exposure. Very few newborns are registered in the HDSS within the first month following their birth, and calculations of neonatal mortality using observation date exposure produces many estimates of zero. Contrarily, 62% of postneonatal mortality and 80% of child mortality estimates are higher when the observation date is used as the beginning of exposure. The results for infant mortality are more evenly split in terms of producing higher estimates with event or observation date exposure.

We calculated the HDSS estimates using individual-level data on deaths and exposure, where individuals contributed exposure time starting from birth or in-migration to the HDSS. For a subset of studies where data was available, we also calculated estimates with exposure time beginning at the date that these events were observed by the HDSS, rather than the dates of the events themselves. Overall, estimates that used exposure time since observation date were lower for neonatal mortality, and slightly higher for postneonatal and child mortality (Figure A-3). This reinforces the above findings of HDSS–DHS differences in mortality under-5, and suggests that the gap between estimates from each source would increase if HDSS estimates used exposure time since observation date.

Table 1 presents the regression models for relative percentage differences between HDSS and DHS subnational region estimates. In the bivariate models for neonatal mortality, the covariates of HDSS pregnancy reporting and African region were strongly associated with the response variable. In HDSSs that use information on pregnancies to follow up on pregnancy outcomes, estimates of neonatal mortality were roughly 15% higher than DHS estimates. While this association disappears in the multivariable model, the effect for Southern African region becomes even stronger. Treating Eastern Africa as the reference category, estimates of neonatal mortality from HDSSs in Southern Africa were approximately 36% lower than DHS. This also affects infant mortality estimation, where HDSSs from Southern Africa had estimates that were approximately 15% lower.

Controlling for all other covariates in the models, the association of HDSS digit preference was in opposite directions for neonatal and postneonatal mortality. Increased digit preference was associated with lower levels of HDSS neonatal mortality and higher levels of postneonatal mortality. In the bivariate models the coefficient of HDSS interview interval was strongly associated with differences in postneonatal mortality. HDSSs with longer interview intervals, and thus less frequent interviews, typically had lower estimates of postneonatal mortality relative to the DHS. The effect was weaker in multivariable models, but indicated a 1% decrease in HDSS estimates of postneonatal mortality for each one month increase in the time between interview rounds.

The coefficient for DHS data quality was negative and moderately associated with the response variable in each bivariate model, as well as the multivariable models for postneonatal and child mortality. For each one-unit improvement in DHS data quality, DHS estimates increased relative to HDSS by around 6%. Malaria endemicity was associated with higher HDSS estimates of postneonatal and infant mortality by approximately 17% and 13% respectively. Many HDSSs have been set up in areas known to have high malaria transmission (Deribew et al. 2016), and therefore may have higher mortality than the surrounding DHS subnational region. However, the effect of malaria on HDSS–DHS differences was not as strong in the multivariable models. The number of average annual births taking place in HDSSs was not associated with differences with DHS in any of the multivariable models. The covariates for DHS TIPS and DHS adult mortality were not included in the multivariable models, as they were not strongly associated with the response variable in any of the bivariate models.

The *R*^2^ value for the child mortality model was lower than that for other models. This indicates that the covariates explained less of the variance observed in HDSS–DHS differences in child mortality. There were stronger associations with the covariates in the neonatal, postneonatal, and infant mortality models, which accounted for roughly one-third of the variation in HDSS–DHS differences.

## 4. Discussion

Contemporary data sources for U5M in sub-Saharan Africa are all suggestive of an age pattern that deviates considerably from the record of high-quality CRVS data from (mostly) high income countries. This atypical age pattern, characterized by higher levels of child mortality relative to infant or U5M, has previously been described for Western African populations (Abdullah et al. 2007; Billewicz and McGregor 1981; Cantrelle 1969; Garenne 1981; McGregor, Billewicz, and Thomson 1961; Pison and Langaney 1985). Our analyses suggest that this phenomenon is not exclusive to Western Africa but also characterizes the estimates for other African regions, albeit to a lesser extent.

Various factors could contribute to a different age pattern of mortality in African populations. Infectious diseases like HIV, malaria, measles, and diarrhea create an environment with relatively high levels of child mortality (Guillot et al. 2012). Mortality from these causes is typically lower in the first six months of life because children are not exposed or acquire passive immunity from their mother through breast milk. Once breastfeeding ends, however, this layer of protection wanes and mortality from these causes increases (Cantrelle and Leridon 1971; Garenne 1981). These factors combined with inadequate diet generate excess mortality at later ages, and thus an older age pattern of U5M (Guillot et al. 2012).

However, it remains possible that this conclusion is compromised by data quality issues for data sources in sub-Saharan Africa (i.e., HDSSs and FBHs). In the absence of well-functioning CRVS there is no gold standard measurement of U5M for many low- and middle-income populations. FBHs collected in periodic surveys have for a long time served as a satisfying stopgap, and they continue to be the primary data source for worldwide estimates of U5M (Alkema and New 2014; Wang et al. 2017). However, FBHs are subject to data quality issues that give reason to regard such estimates with caution (Dwyer-Lindgren et al. 2013; Pullum and Becker 2014; Schoumaker 2014).

The omission of births or deaths from FBHs is a serious issue that occurs more often when the child dies at an early age (Pullum et al. 2013). There is also evidence of misclassification of stillbirths and neonatal deaths in FBHs that could create upward bias in estimates of neonatal mortality. One study in Malawi found that 20% of 365 neonatal deaths in FBHs were classified as stillbirths by verbal autopsy questionnaires (Liu et al. 2016). This tendency to misclassify stillbirths as early neonatal deaths was corroborated by another study from Guinea Bissau (Helleringer et al. 2020). In surveys where recent births or deaths are subjected to more detailed data collection, it has been found that data collectors may displace births or deaths outside of this reporting window in order to reduce their workload (Pullum and Becker 2014; Schoumaker 2011). Disproportionate reporting of deaths at certain central ages such as 1 month and 12 months is also common in FBHs (Pullum et al. 2013; Pullum and Staveteig 2017). If such heaping was primarily the result of upward rounding, this practice could distort FBH estimates of neonatal, postneonatal, and child mortality, and thus the age pattern of U5M.

The quality of early childhood mortality data in HDSSs is also questionable. Some of the earliest research on age patterns of U5M in West Africa called attention to the issue of under-registration of early deaths in HDSSs (Cantrelle 1969; McGregor and Williams1979). The severity of this underestimation was thought to be reduced by the follow-up of pregnancies (Cantrelle and Leridon 1971); however, the imperfect and incomplete nature of this information meant that early deaths were still likely under-counted (Garenne 1981). In our multivariable regression analysis, conducting pregnancy reporting was not strongly associated with higher estimates of neonatal mortality (relative to DHS). This is perhaps due to the heterogeneity in pregnancy reporting completeness across HDSSs (Waiswa et al. 2019). There are also procedural inconsistencies regarding the use of proxy respondents and male interviewers, which have been shown to negatively impact pregnancy reporting completeness (Kadobera et al. 2017). Collecting information on pregnancies and reliably detecting neonatal deaths remain some of the most challenging issues in HDSSs (Sankoh and Byass 2012).

It is thus not surprising that HDSS estimates of neonatal mortality were approximately 8% and 14% lower than subnational estimates from DHS and MICS, respectively. The DHS and MICS early mortality estimates appear more realistic, with ratios of postneonatal-to-neonatal mortality that were consistent with the model based on high-quality CRVS data. However, it is reasonable to conclude that estimation of early child mortality poses problems for both FBHs and HDSSs, and further validation is needed to better understand bias in each source. In general, overestimation in DHS and underestimation in HDSS could contribute to some of the large differences in estimates of neonatal mortality, though it is also important to note that there is considerable heterogeneity in the level of recorded neonatal mortality across HDSSs, and it remains difficult to adjudicate whether this is due to the epidemiological context, data collection practices, or a combination of both.

Our comparison of HDSS and subnational DHS and MICS estimates of child mortality was more conclusive, and suggests that FBH estimates are biased downward. On average, HDSS estimates of child mortality were approximately 7% higher than DHS subnational estimates and 6% higher than MICS. Accounting for the left censoring of HDSS data increased the magnitude of this difference even further. In theory, HDSSs should be well-placed to prospectively track child mortality. The longer a child survives, the more likely they are to be added to the HDSS household roster, which in turn prompts fieldworkers to inquire about their vital and residency statuses at each follow-up interview. Thus, a prospective surveillance system is well-suited to accurately measure late child mortality. By contrast, FBHs are vulnerable to retrospective reporting errors. These include the omission of past births or deaths, and date misreporting errors when the child’s date of birth or age at death are not accurately reported (Pullum and Becker 2014). This proposition is supported by our finding that DHS markers of low data quality were correlated with a larger disparity with the postneonatal and child mortality estimates from HDSSs.

We identified other factors correlated with the disparity between HDSS and DHS estimates of U5M in the regression analysis. HDSS digit preference in day of death reporting was associated with lower neonatal and higher postneonatal mortality. This suggests that HDSS underestimation of neonatal mortality is not solely due to poor registration of newborns, but also rounding errors in the reported ages at death. Estimates of neonatal mortality for HDSSs located in Southern Africa were also much lower than DHS estimates. It could be that HDSS estimates for this region are affected by downward bias that was not well-captured by the covariates in the model. A more detailed examination of the fieldwork procedures and data quality of HDSSs in this region is recommended.

This study has several important limitations. First, HDSSs produce mortality estimates for localized areas that are generally much smaller than DHS or MICS subnational regions. HDSS data is not designed to be representative of the broader region, and some differences in HDSS and FBH mortality estimates could be attributable to geographic variations. For example, higher values of HDSS child mortality could be due to HDSSs being located in areas that have higher mortality relative to the general population (Deribew et al. 2016). Conversely, it has also been suggested that HDSS residents may benefit from living in a testing ground for public health interventions (Sankoh and Byass 2012).

**Figure A-4:**
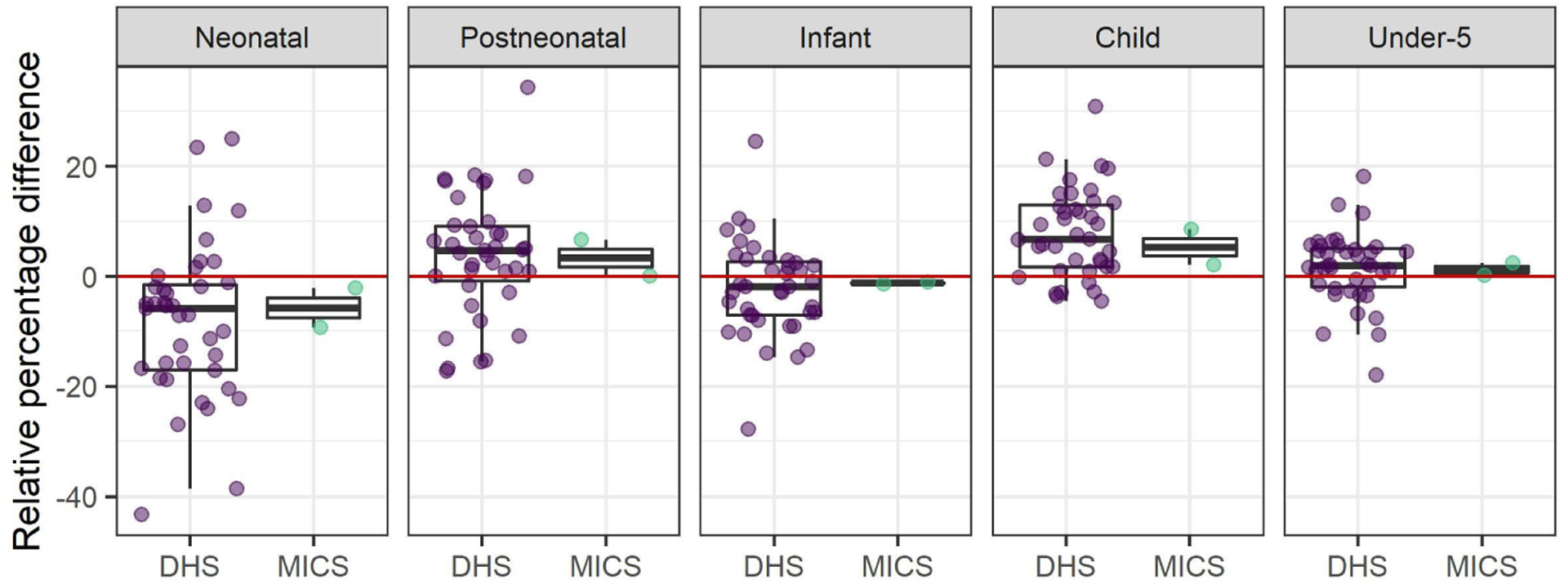
HDSS-FBH differences for 10-year periods *Note:* Relative percentage differences between HDSS and DHS/MICS subnational region mortality estimates for overlapping 10-year periods. There are 39 overlapping periods for HDSS and DHS, and 2 for HDSS and MICS.

**Figure A-5:**
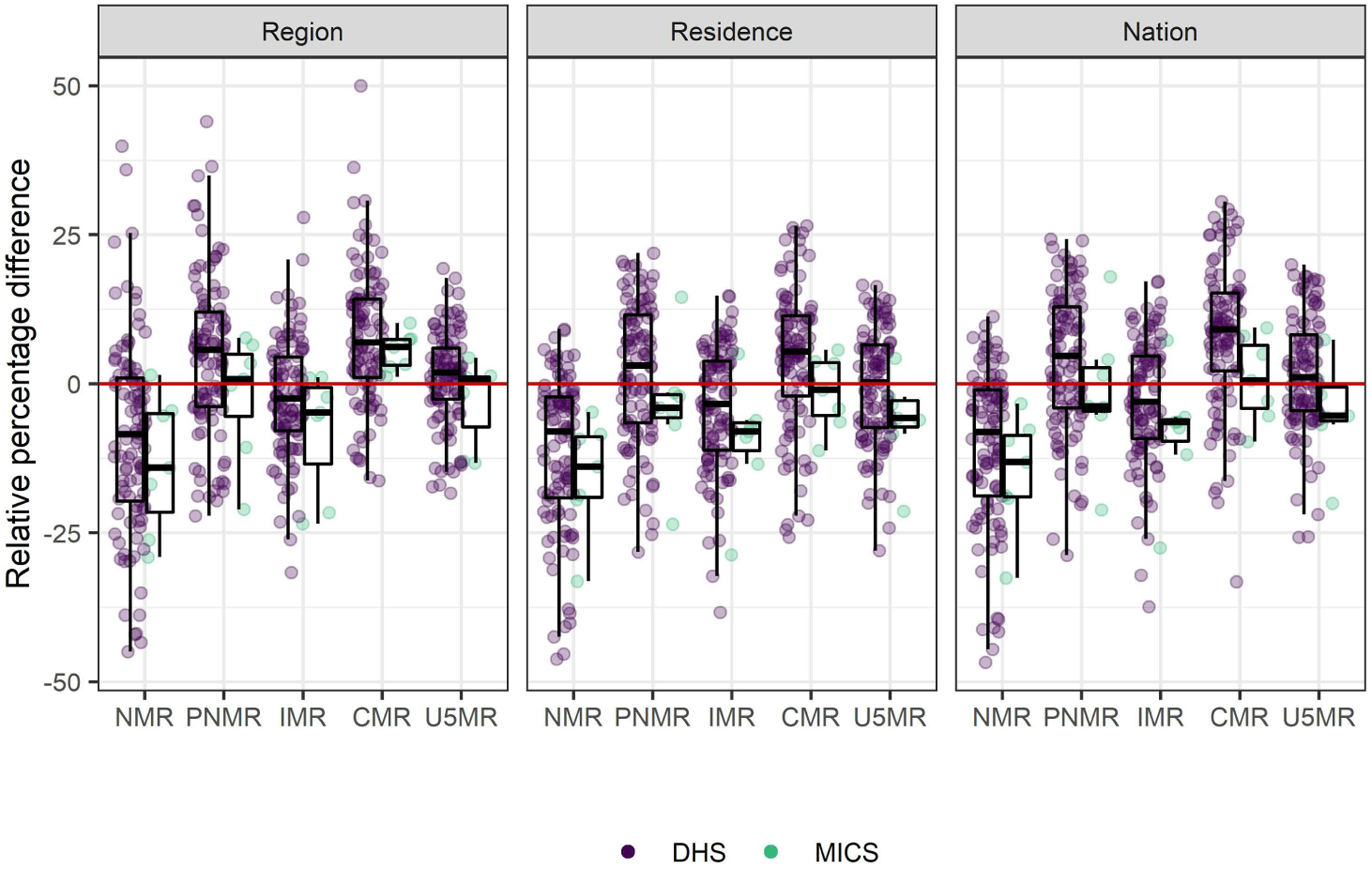
Differences between HDSS and FBH subnational region, residence, and national estimates *Note:* Relative percentage differences between HDSS and DHS/MICS. The subnational region differences are the same as those displayed in Figure 3. HDSS differences with DHS/MICS residence (urban/rural) and national estimates have been included for comparison. HDSS were categorized as urban or rural based on the residence of the majority of the study population.

There is also the issue of small sample sizes when stratifying DHS and MICS estimates by subnational region. The DHS typically reports mortality estimates for sub-populations in 10-year periods due to concerns regarding sample size and displacement of events out of the most recent 5-year period (Pullum and Becker 2014). We used 5-year periods in our analysis, but found that the main conclusions regarding HDSS-FBH differences remained unchanged with the use of a 10-year window (Figure A-4). We also compared HDSS mortality estimates with national and residence (urban/rural) level FBH estimates, which have larger sample sizes (Figure A-5). In this investigation, the magnitude and direction of differences between HDSS and DHS estimates were similar to the subnational region comparison. The results changed slightly for MICS, where the median difference for national and residence level estimates with HDSS was negative for postneonatal mortality and close to zero for child mortality. It is important to interpret individual differences between HDSSs and FBHs cautiously due to the variation in sample sizes and relative rarity of mortality as an event. Nevertheless, the aggregate differences and regression analysis provide useful insight into discrepancies between DHS and HDSS estimates, and the contextual and data attributes that may explain them.

## 5. Conclusion

Estimates of U5M for sub-Saharan Africa heavily rely on a combination of FBH data from surveys and model age patterns (UN IGME 2018; Wang et al. 2017). This practice is problematic for two reasons. First, the comparison of African HDSS and FBH data shows that FBH estimates of U5M may be affected by downward bias. Second, our analyses suggest that the age pattern of U5M is atypical and not well represented by existing model life tables. More specifically, our findings indicate that sub-Saharan African populations are characterized by relatively high levels of child mortality for a given level of infant mortality, and this could invalidate the use of model age patterns derived from both contemporary and historical data of populations with high-quality CRVS. This conclusion comes with the caveat that neither of our data sources (FBH or HDSS) represent a gold standard measurement of U5M. Ultimately, there is a need for accurate primary data on U5M, and such data will be essential to reaching targets for its reduction.

## Supplementary Material

Supplementary Tables

Data and ReadMe Files

## Figures and Tables

**Table 1:** Linear regression on relative differences in HDSS and DHS subnational region estimates of mortality under-5

	Dependent variable: Relative percentage difference in HDSS and DHS mortality estimates			
	Neonatal		Postneonatal	
	Bivariate models (SE); p-value	Multivariable model (SE); p-value	Bivariate models (SE); p-value	Multivariable model (SE); p-value
HDSS avg. births	0.20 (1.52); p = 0.89	3.37 (2.52); p = 0.18	5.08 (1.14); p < 0.01	2.14 (2.00); p = 0.29
HDSS digit preference	7.52 (7.13); p = 0.29	−21.92 (9.67); p = 0.03	13.54 (5.73); p = 0.02	27.11 (7.66); p < 0.01
HDSS interview interval	−0.02 (0.40); p = 0.96	1.82 (0.73); p = 0.02	−1.02 (0.31); p < 0.01	−1.18 (0.58); p = 0.05
HDSS preg. reporting	14.90 (4.47); p < 0.01	3.93 (5.59); p = 0.48	0.86 (3.87); p = 0.82	0.71 (4.43); p = 0.87
DHS adult mortality	−50.32 (36.92); p = 0.18		−9.76 (24.89); p = 0.70	
DHS data quality	−6.65 (3.50); p = 0.06	1.64 (3.94); p = 0.67	−6.04 (2.87); p = 0.04	−6.14 (3.12); p = 0.05
DHS TIPS (ref. = 0–4 years)				
5–9 years	2.49 (3.29); p = 0.45		−3.02 (2.69); p = 0.26	
Malaria endemicity	7.03 (8.34); p = 0.40	−0.74 (8.51); p = 0.93	16.96 (6.49); p = 0.01	11.15 (6.74); p = 0.10
African region (ref. = Eastern)				
Southern	−26.62 (6.12); p < 0.01	−36.09 (8.27); p < 0.01	−11.25 (5.52); p = 0.04	2.23 (6.54); p = 0.73
Western	2.77 (3.19); p = 0.39	2.57 (4.83); p = 0.60	−1.63 (2.88); p = 0.57	−4.32 (3.82); p = 0.26
Constant	−8.94 (1.62); p < 0.01	−40.47 (20.89); p = 0.06	4.81 (1.33); p < 0.01	−10.47 (16.54); p = 0.53
Observations		83		83
R^2^		0.32		0.35
Adjusted R^2^		0.25		0.28
Residual Std. Error (df = 74)		13.91		11.03
F Statistic (df = 8; 74)		4.39 (p<0.01)		4.90 (p<0.01)

	Dependent variable: Relative percentage difference in HDSS and DHS mortality estimates			
	Infant		Child	
	Bivariate models (SE); p-value	Multivariable model (SE); p-value	Bivariate models (SE); p-value	Multivariable model (SE); p-value
HDSS avg. births	2.37 (0.89); p = 0.01	1.78 (1.35); p = 0.19	3.12 (1.01); p < 0.01	1.61 (2.09); p = 0.44
HDSS digit preference	10.57 (4.22); p = 0.01	5.37 (5.17); p = 0.30	−5.55 (4.97); p = 0.27	−0.73 (8.02); p = 0.93
HDSS interview interval	−0.39 (0.24); p = 0.11	0.25 (0.39); p = 0.52	−0.79 (0.27); p < 0.01	−0.74 (0.61); p = 0.23
HDSS preg. reporting	6.40 (2.79); p = 0.02	1.44 (2.99); p = 0.63	−2.77 (3.28); p = 0.40	−0.20 (4.64); p = 0.97
DHS adult mortality	−20.86 (20.77); p = 0.32		2.62 (22.98); p = 0.91	
DHS data quality	−5.40 (2.09); p = 0.01	−2.23 (2.11); p = 0.29	−4.89 (2.44); p = 0.05	−6.45 (3.27); p = 0.05
DHS TIPS (ref. = 0–4 years)				
5–9 years	−0.27 (2.00); p = 0.89		−3.50 (2.27); p = 0.13	
Malaria endemicity	12.76 (4.34); p < 0.01	7.11 (4.55); p = 0.12	3.29 (6.21); p = 0.60	1.58 (7.06); p = 0.82
African region (ref. = Eastern)				
Southern	−17.94 (3.70); p < 0.01	−15.27 (4.42); p < 0.01	1.46 (4.78); p = 0.76	5.99 (6.86); p = 0.39
Western	−0.28 (1.93); p = 0.89	−2.62 (2.58); p = 0.31	−1.24 (2.49); p = 0.62	0.89 (4.01); p = 0.82
Constant	−2.39 (0.98); p = 0.02	−18.57 (11.18); p = 0.10	7.80 (1.13); p < 0.01	2.59 (17.32); p = 0.88
Observations		83		83
R^2^		0.35		0.15
Adjusted R^2^		0.28		0.06
Residual Std. Error (df = 74)		7.45		11.55
F Statistic (df = 8; 74)		4.91 (p < 0.01)		1.68 (p = 0.12)

## Footnotes

3
http://www.indepth-ishare.org

4
https://dhsprogram.com/data/available-datasets.cfm

5
http://mics.unicef.org/surveys

6
https://www.mortality.org/

7 https://malariaatlas.org/.

## Appendix

**Table A-1:** Ratios of child-to-infant mortality for all data sources and African regions

Region	Source	Median	IQR	P-value
All	HMD	0.34	(0.27, 0.45)	< 0.01
	DHS	0.61	(0.49, 0.74)	-
	MICS	0.55	(0.41, 0.72)	0.08
	HDSS	0.79	(0.63, 1.03)	< 0.01
Southern	DHS	0.38	(0.30, 0.45)	-
	MICS	0.35	(0.32, 0.40)	0.71
	HDSS	0.74	(0.62, 1.03)	< 0.01
Eastern	DHS	0.57	(0.46, 0.65)	-
	MICS	0.54	(0.43, 0.66)	0.86
	HDSS	0.74	(0.51, 0.87)	< 0.01
Central	DHS	0.71	(0.57, 0.83)	-
	MICS	0.58	(0.44, 0.71)	0.14
Western-All	DHS	0.66	(0.53, 0.80)	-
	MICS	0.65	(0.45, 0.74)	0.36
	HDSS	0.93	(0.77, 1.17)	< 0.01
Western-Coastal	DHS	0.65	(0.60, 0.73)	-
	MICS	0.65	(0.60, 0.72)	0.89
	HDSS	0.85	(0.79, 0.98)	< 0.01
Western-Sahelian	DHS	0.68	(0.51, 0.89)	-
	MICS	0.58	(0.29, 0.84)	0.21
	HDSS	0.96	(0.74, 1.33)	0.02

**Table A-2:** Ratios of postneonatal-to-neonatal mortality for all data sources and African regions

Region	Source	Median	IQR	P-value
All	DHS	0.85	(0.70, 1.07)	-
	MICS	0.98	(0.77, 1.37)	0.06
	HDSS	1.41	(0.98, 2.28)	< 0.01
Southern	DHS	0.91	(0.80, 1.15)	-
	MICS	2.10	(1.41, 3.06)	0.01
	HDSS	3.19	(2.72, 4.41)	< 0.01
Eastern	DHS	0.91	(0.65, 1.05)	-
	MICS	1.13	(0.96, 1.47)	< 0.01
	HDSS	1.26	(0.98, 2.18)	< 0.01
Central	DHS	1.05	(0.83, 1.24)	-
	MICS	0.92	(0.77, 1.12)	0.49
Western-All	DHS	0.80	(0.65, 0.98)	-
	MICS	0.78	(0.55, 0.97)	0.44
	HDSS	1.16	(0.94, 1.79)	< 0.01
Western-Coastal	DHS	0.85	(0.79, 1.04)	-
	MICS	0.78	(0.62 1.05)	0.58
	HDSS	1.16	(1.07, 1.52)	0.09
Western-Sahelian	DHS	0.71	(0.64, 0.91)	-
	MICS	0.74	(0.50, 0.85)	0.40
	HDSS	1.19	(0.94, 1.81)	< 0.01

**Table A-3:** Differences in HDSS and FBH estimates of mortality displayed in Figure 3

Mortality	Source	Median	IQR
Neonatal	DHS	−8.48	(−19.65, 1.01)
	MICS	−14.10	(−21.53, −4.96)
Postneonatal	DHS	5.68	(−3.85, 12.06)
	MICS	0.75	(−5.46, −4.97)
Infant	DHS	−2.52	(−7.90, 4.50)
	MICS	−4.83	(−13.48, −0.62)
Child	DHS	6.90	(1.10, 14.24)
	MICS	6.14	(3.07, 7.46)
Under-5	DHS	1.85	(−2.54, 6.00)
	MICS	0.64	(−7.23, 1.18)
