# Supplementary material for: Age patterns of under-5 mortality in sub-Saharan Africa during 1990–2018: A comparison of estimates from demographic surveillance with full birth histories and the historic record
Source: Demogr Res. Author manuscript; Available in PMC 2022 Apr 1. (PMC8974662; doi:10.4054/demres.2021.44.18)
Supplement: Supplementary Tables [file NIHMS1702795-supplement-Supplementary_Tables.pdf]

Table S-1: HDSS mortality estimates for Figures 1 and 2.

			Neonatal				Postneonatal				Child			
Country	HDSS	Period	Deaths	PY	q(x)	CI	Deaths	PY	q(x)	CI	Deaths	PY	q(x)	CI
Burkina Faso	Nanoro	2011-2015	94	747	0.010	(0.008, 0.012)	145	9260	0.014	(0.012, 0.017)	241	39766	0.024	(0.021, 0.027)
Burkina Faso	Nouna	2001-2005	142	796	0.014	(0.012, 0.016)	481	9564	0.045	(0.042, 0.049)	805	38774	0.079	(0.074, 0.084)
Burkina Faso	Nouna	2006-2010	130	1038	0.010	(0.008, 0.011)	405	12322	0.030	(0.027, 0.033)	801	48654	0.062	(0.058, 0.066)
Burkina Faso	Nouna	2011-2015	180	1248	0.011	(0.010, 0.013)	399	15047	0.024	(0.022, 0.027)	801	59033	0.052	(0.049, 0.055)
Burkina Faso	Ouagadougou	2011-2015	136	887	0.012	(0.010, 0.014)	138	11541	0.011	(0.009, 0.013)	239	52475	0.018	(0.016, 0.020)
Cote d'Ivoire	Taabo	2012-2016	116	586	0.015	(0.013, 0.018)	261	7329	0.032	(0.029, 0.036)	397	29905	0.050	(0.046, 0.055)
Ethiopia	Arba Minch	2011-2015	66	752	0.007	(0.005, 0.009)	137	8502	0.015	(0.012, 0.017)	155	36488	0.017	(0.015, 0.020)
Ethiopia	Dabat	2011-2015	17	441	0.003	(0.002, 0.005)	80	6151	0.012	(0.010, 0.015)	103	30107	0.014	(0.011, 0.017)
Ethiopia	Gilgel Gibe	2006-2010	380	642	0.044	(0.040, 0.049)	263	7326	0.032	(0.029, 0.036)	274	31013	0.035	(0.031, 0.039)
Ethiopia	Gilgel Gibe	2011-2015	332	713	0.035	(0.032, 0.039)	239	8532	0.025	(0.023, 0.029)	213	34754	0.024	(0.021, 0.027)
Ethiopia	Harar	2012-2016	14	233	0.005	(0.003, 0.008)	15	2989	0.005	(0.003, 0.008)	16	13709	0.005	(0.003, 0.008)
Ethiopia	Kersa	2012-2016	342	989	0.026	(0.024, 0.029)	338	11999	0.026	(0.023, 0.028)	555	48064	0.045	(0.041, 0.048)
Ethiopia	Kilite Awlaelo	2010-2014	129	555	0.018	(0.015, 0.021)	67	6290	0.010	(0.008, 0.012)	83	28923	0.012	(0.010, 0.015)
Gambia	Farafenni	2001-2005	92	437	0.016	(0.013, 0.020)	166	5291	0.029	(0.025, 0.033)	283	21651	0.050	(0.045, 0.056)
Gambia	Farafenni	2006-2010	95	606	0.012	(0.010, 0.015)	121	7414	0.015	(0.013, 0.018)	191	29825	0.025	(0.021, 0.028)
Gambia	Farafenni	2011-2015	140	674	0.016	(0.013, 0.019)	126	8226	0.014	(0.012, 0.017)	172	35218	0.019	(0.017, 0.022)
Ghana	Dodowa	2007-2011	103	1018	0.008	(0.006, 0.009)	125	12627	0.009	(0.008, 0.011)	247	58881	0.017	(0.015, 0.019)
Ghana	Kintampo	2010-2014	424	1652	0.019	(0.018, 0.021)	302	20704	0.013	(0.012, 0.015)	520	84352	0.024	(0.022, 0.026)
Ghana	Navrongo	2000-2004	295	783	0.028	(0.026, 0.032)	476	9237	0.046	(0.043, 0.050)	565	37144	0.059	(0.055, 0.063)
Ghana	Navrongo	2005-2009	224	740	0.023	(0.020, 0.026)	250	8783	0.026	(0.023, 0.029)	352	36404	0.038	(0.034, 0.042)
Ghana	Navrongo	2010-2014	128	789	0.012	(0.010, 0.015)	133	9379	0.013	(0.011, 0.015)	222	38444	0.023	(0.020, 0.026)
Kenya	Kombewa	2011-2015	90	955	0.007	(0.006, 0.009)	298	13470	0.020	(0.018, 0.023)	559	80230	0.029	(0.027, 0.032)
Kenya	Mbita	2011-2015	11	222	0.004	(0.002, 0.007)	41	2801	0.013	(0.010, 0.018)	125	19298	0.028	(0.023, 0.033)
Kenya	Nairobi	2006-2010	150	758	0.015	(0.013, 0.018)	352	9195	0.035	(0.031, 0.038)	233	34023	0.026	(0.023, 0.029)
Kenya	Nairobi	2011-2015	199	674	0.022	(0.020, 0.026)	255	8242	0.028	(0.025, 0.032)	147	32406	0.018	(0.015, 0.021)
Malawi	Karonga	2003-2007	140	475	0.022	(0.019, 0.026)	161	5594	0.026	(0.023, 0.030)	193	21374	0.034	(0.030, 0.039)

Malawi	Karonga	2008-2012	131	503	0.020	(0.017, 0.023)	129	6001	0.020	(0.017, 0.023)	174	25448	0.027	(0.023, 0.031)
Malawi	Karonga	2013-2017	156	469	0.025	(0.022, 0.029)	39	5713	0.006	(0.005, 0.009)	67	25429	0.010	(0.008, 0.013)
Mozambique	Chokwe	2012-2016	155	1085	0.011	(0.009, 0.013)	275	13876	0.018	(0.016, 0.020)	652	61571	0.041	(0.038, 0.044)
Nigeria	Cross River	2013-2017	4	16	0.019	(0.007, 0.049)	12	1326	0.009	(0.005, 0.016)	36	11849	0.013	(0.009, 0.018)
Senegal	Bandafassi	2002-2006	119	195	0.046	(0.039, 0.054)	105	2230	0.043	(0.036, 0.051)	181	8162	0.082	(0.072, 0.093)
Senegal	Bandafassi	2007-2011	55	192	0.022	(0.017, 0.028)	101	2266	0.040	(0.033, 0.048)	180	8993	0.076	(0.067, 0.086)
Senegal	Bandafassi	2012-2016	63	190	0.025	(0.020, 0.032)	62	2249	0.025	(0.020, 0.032)	97	9185	0.041	(0.034, 0.049)
Senegal	Mlomp	2002-2006	16	67	0.018	(0.011, 0.029)	17	743	0.020	(0.013, 0.032)	23	3317	0.028	(0.019, 0.041)
Senegal	Mlomp	2007-2011	23	67	0.026	(0.018, 0.038)	9	780	0.011	(0.006, 0.020)	12	3238	0.015	(0.009, 0.026)
Senegal	Mlomp	2012-2016	13	62	0.016	(0.009, 0.027)	5	779	0.006	(0.003, 0.014)	6	3516	0.007	(0.003, 0.015)
Senegal	Niakhar	2002-2006	128	509	0.019	(0.016, 0.023)	202	5932	0.031	(0.027, 0.035)	419	22315	0.070	(0.064, 0.076)
Senegal	Niakhar	2007-2011	59	568	0.008	(0.006, 0.010)	109	6944	0.014	(0.012, 0.017)	219	27136	0.031	(0.027, 0.035)
Senegal	Niakhar	2012-2016	97	606	0.012	(0.010, 0.015)	93	7353	0.012	(0.010, 0.014)	181	30859	0.023	(0.020, 0.026)
South Africa	AHRI	2003-2007	68	692	0.008	(0.006, 0.009)	310	8496	0.033	(0.030, 0.037)	266	34595	0.030	(0.027, 0.033)
South Africa	AHRI	2008-2012	26	673	0.003	(0.002, 0.004)	234	8637	0.025	(0.022, 0.028)	142	39341	0.014	(0.012, 0.017)
South Africa	AHRI	2013-2017	19	576	0.003	(0.002, 0.004)	67	7709	0.008	(0.006, 0.010)	79	38606	0.008	(0.007, 0.010)
South Africa	Agincourt	2003-2007	88	684	0.010	(0.008, 0.012)	281	8069	0.032	(0.028, 0.035)	228	33339	0.027	(0.024, 0.030)
South Africa	Agincourt	2008-2012	127	859	0.011	(0.010, 0.013)	268	10329	0.024	(0.021, 0.027)	195	43646	0.018	(0.015, 0.020)
South Africa	Agincourt	2013-2017	74	936	0.006	(0.005, 0.008)	106	11660	0.008	(0.007, 0.010)	119	53194	0.009	(0.007, 0.011)
South Africa	Dikgale	2002-2006	3	62	0.004	(0.001, 0.011)	8	736	0.010	(0.005, 0.020)	11	3080	0.014	(0.008, 0.025)
South Africa	Dikgale	2007-2011	0	109	0.000	(0.000, 0.003)	9	1533	0.005	(0.003, 0.010)	22	7503	0.011	(0.007, 0.017)
South Africa	Dikgale	2012-2016	4	250	0.001	(0.000, 0.003)	13	3070	0.004	(0.002, 0.007)	28	15871	0.007	(0.005, 0.010)
Tanzania	Ifakara	2000-2004	365	935	0.029	(0.027, 0.032)	561	11093	0.046	(0.042, 0.049)	643	43233	0.057	(0.053, 0.061)
Tanzania	Ifakara	2005-2009	486	1155	0.032	(0.029, 0.035)	547	13800	0.036	(0.033, 0.039)	550	56579	0.038	(0.035, 0.041)
Tanzania	Ifakara	2010-2014	527	1369	0.029	(0.027, 0.032)	420	16661	0.023	(0.021, 0.025)	502	76950	0.026	(0.024, 0.028)
Tanzania	Magu	2003-2007	52	328	0.012	(0.009, 0.016)	206	4083	0.046	(0.040, 0.052)	196	17897	0.042	(0.037, 0.048)
Tanzania	Magu	2008-2012	9	325	0.002	(0.001, 0.004)	126	4231	0.028	(0.023, 0.033)	176	21253	0.033	(0.029, 0.038)
Tanzania	Rufiji	2000-2004	348	1057	0.025	(0.023, 0.028)	621	12259	0.046	(0.042, 0.049)	571	44767	0.048	(0.045, 0.052)
Tanzania	Rufiji	2005-2009	285	1082	0.020	(0.018, 0.022)	400	12755	0.029	(0.026, 0.031)	574	49059	0.044	(0.041, 0.048)
Tanzania	Rufiji	2010-2014	223	977	0.017	(0.015, 0.020)	242	11948	0.019	(0.016, 0.021)	353	53507	0.026	(0.024, 0.029)
Uganda	Iganga Mayuge	2007-2011	276	762	0.027	(0.024, 0.031)	370	9503	0.035	(0.032, 0.039)	512	40268	0.049	(0.045, 0.053)

Uganda	Iganga Mayuge	2012-2016	301	781	0.029	(0.026, 0.032)	259	9960	0.024	(0.021, 0.027)	452	46559	0.039	(0.035, 0.042)

Note -- PY: person-years, CI: 95% confidence intervals calculated using Taylor linearisation.

Table S-2: DHS mortality estimates for Figures 1 and 2.

			Neonatal				Postneontal				Child			
Country	Survey year	Period	Deaths	PY	q(x)	CI	Deaths	PY	q(x)	CI	Deaths	PY	q(x)	CI
Angola	2015	2005-2009	268	838	0.024	(0.020, 0.029)	351	9557	0.033	(0.028, 0.038)	332	32753	0.039	(0.033, 0.045)
Angola	2015	2010-2014	327	1020	0.024	(0.021, 0.028)	260	11878	0.020	(0.017, 0.023)	277	46011	0.023	(0.019, 0.028)
Benin	2012	2002-2006	321	969	0.025	(0.022, 0.029)	305	11598	0.024	(0.021, 0.027)	344	42029	0.031	(0.028, 0.035)
Benin	2012	2007-2011	308	1034	0.023	(0.019, 0.026)	234	12019	0.018	(0.015, 0.020)	330	50497	0.026	(0.022, 0.029)
Benin	2017	2007-2011	415	870	0.036	(0.032, 0.040)	342	10194	0.031	(0.027, 0.034)	470	36655	0.049	(0.044, 0.054)
Benin	2017	2012-2016	402	1013	0.030	(0.026, 0.034)	327	11692	0.025	(0.022, 0.029)	479	45952	0.040	(0.036, 0.044)
Burkina Faso	2010	2000-2004	598	1086	0.041	(0.037, 0.045)	731	12783	0.051	(0.047, 0.056)	1033	43667	0.087	(0.080, 0.094)
Burkina Faso	2010	2005-2009	430	1155	0.028	(0.025, 0.031)	539	13281	0.037	(0.033, 0.041)	851	52761	0.062	(0.056, 0.067)
Burundi	2010	2000-2004	273	460	0.045	(0.037, 0.052)	365	5236	0.062	(0.054, 0.070)	322	17278	0.070	(0.061, 0.079)
Burundi	2010	2005-2009	234	577	0.031	(0.026, 0.035)	206	6723	0.028	(0.023, 0.032)	230	25054	0.035	(0.030, 0.040)
Burundi	2016	2006-2010	335	921	0.028	(0.024, 0.031)	304	10688	0.026	(0.023, 0.029)	370	39101	0.036	(0.032, 0.040)
Burundi	2016	2011-2015	300	992	0.023	(0.019, 0.026)	313	11732	0.024	(0.021, 0.028)	364	47611	0.030	(0.026, 0.034)
Cameroon	2011	2001-2005	354	780	0.034	(0.030, 0.038)	450	9105	0.045	(0.039, 0.050)	571	32316	0.065	(0.058, 0.072)
Cameroon	2011	2006-2010	368	889	0.031	(0.027, 0.035)	357	10200	0.032	(0.028, 0.036)	594	39163	0.058	(0.053, 0.063)
Cameroon	2018	2008-2012	281	664	0.032	(0.026, 0.037)	236	7786	0.028	(0.023, 0.032)	307	27289	0.043	(0.038, 0.049)
Cameroon	2018	2013-2017	272	731	0.028	(0.023, 0.033)	189	8620	0.020	(0.017, 0.023)	286	35071	0.032	(0.027, 0.036)
Chad	2014	2004-2008	738	1421	0.039	(0.035, 0.043)	1014	16770	0.054	(0.046, 0.062)	1245	56263	0.081	(0.072, 0.089)
Chad	2014	2009-2013	620	1392	0.034	(0.029, 0.038)	686	16012	0.039	(0.035, 0.043)	1069	68759	0.060	(0.055, 0.065)
Comoros	2012	2002-2006	80	220	0.028	(0.020, 0.035)	40	2622	0.014	(0.008, 0.020)	23	9769	0.009	(0.004, 0.014)
Comoros	2012	2007-2011	75	242	0.023	(0.017, 0.030)	36	2818	0.012	(0.006, 0.017)	37	11506	0.013	(0.007, 0.018)
Congo	2011	2001-2005	217	585	0.028	(0.022, 0.035)	223	6689	0.030	(0.024, 0.037)	288	23856	0.046	(0.038, 0.054)
Congo	2011	2006-2010	209	735	0.022	(0.016, 0.027)	161	8485	0.017	(0.013, 0.021)	219	31873	0.026	(0.022, 0.031)
Congo (DR)	2013	2003-2007	502	1151	0.033	(0.028, 0.038)	610	13232	0.042	(0.037, 0.046)	645	45794	0.053	(0.047, 0.059)

Congo (DR)	2013	2008-2012	524	1423	0.028	(0.024, 0.031)	552	16352	0.031	(0.027, 0.034)	718	61711	0.045	(0.040, 0.050)
Cote d'Ivoire	2012	2002-2006	324	519	0.047	(0.039, 0.054)	267	6105	0.039	(0.033, 0.046)	268	22164	0.046	(0.039, 0.053)
Cote d'Ivoire	2012	2007-2011	293	586	0.038	(0.032, 0.044)	220	6670	0.030	(0.025, 0.035)	270	26288	0.040	(0.034, 0.046)
Ethiopia	2011	2001-2005	555	855	0.049	(0.042, 0.055)	463	10101	0.041	(0.035, 0.048)	469	36372	0.049	(0.040, 0.058)
Ethiopia	2011	2006-2010	424	850	0.038	(0.032, 0.043)	239	9798	0.022	(0.018, 0.026)	289	42828	0.027	(0.022, 0.031)
Ethiopia	2016	2006-2010	499	803	0.046	(0.039, 0.054)	299	9607	0.028	(0.023, 0.034)	217	36530	0.023	(0.018, 0.028)
Ethiopia	2016	2011-2015	299	766	0.029	(0.024, 0.035)	183	8984	0.019	(0.014, 0.023)	182	39570	0.018	(0.013, 0.023)
Gabon	2012	2002-2006	122	386	0.024	(0.016, 0.032)	106	4489	0.022	(0.014, 0.029)	83	17413	0.019	(0.013, 0.024)
Gabon	2012	2007-2011	163	471	0.026	(0.020, 0.032)	86	5414	0.015	(0.010, 0.019)	120	21073	0.022	(0.016, 0.028)
Gambia	2013	2003-2007	224	515	0.033	(0.026, 0.039)	95	5978	0.014	(0.011, 0.018)	130	20728	0.024	(0.018, 0.030)
Gambia	2013	2008-2012	178	613	0.022	(0.018, 0.026)	94	7060	0.012	(0.007, 0.017)	133	27769	0.019	(0.014, 0.024)
Ghana	2014	2004-2008	183	408	0.034	(0.027, 0.040)	117	4932	0.022	(0.017, 0.026)	139	18661	0.029	(0.023, 0.035)
Ghana	2014	2009-2013	171	447	0.029	(0.023, 0.034)	70	5172	0.012	(0.009, 0.016)	90	21085	0.017	(0.013, 0.021)
Guinea	2012	2002-2006	332	515	0.048	(0.042, 0.055)	266	6186	0.039	(0.034, 0.044)	373	21945	0.064	(0.055, 0.073)
Guinea	2012	2007-2011	239	540	0.033	(0.028, 0.039)	233	6185	0.034	(0.028, 0.040)	363	25228	0.056	(0.048, 0.063)
Guinea	2018	2008-2012	262	572	0.034	(0.029, 0.040)	216	6696	0.029	(0.025, 0.034)	290	23315	0.047	(0.041, 0.053)
Guinea	2018	2013-2017	262	605	0.033	(0.028, 0.037)	279	7221	0.035	(0.029, 0.040)	335	29447	0.044	(0.038, 0.050)
Kenya	2014	2004-2008	490	1594	0.023	(0.020, 0.026)	418	18983	0.020	(0.017, 0.023)	329	72053	0.018	(0.015, 0.020)
Kenya	2014	2009-2013	474	1611	0.022	(0.019, 0.025)	349	19100	0.017	(0.014, 0.019)	258	82288	0.012	(0.010, 0.015)
Lesotho	2014	2004-2008	93	211	0.033	(0.025, 0.042)	130	2440	0.048	(0.036, 0.060)	52	8990	0.022	(0.016, 0.029)
Lesotho	2014	2009-2013	105	236	0.034	(0.026, 0.041)	79	2698	0.027	(0.019, 0.034)	70	10554	0.026	(0.018, 0.034)
Liberia	2013	2003-2007	322	583	0.041	(0.032, 0.051)	333	6794	0.044	(0.037, 0.051)	312	23060	0.051	(0.041, 0.060)
Liberia	2013	2008-2012	197	567	0.026	(0.021, 0.032)	202	6560	0.028	(0.023, 0.033)	277	27961	0.039	(0.033, 0.045)
Malawi	2010	2000-2004	669	1395	0.036	(0.033, 0.040)	843	16160	0.047	(0.043, 0.051)	1009	55084	0.068	(0.063, 0.074)
Malawi	2010	2005-2009	629	1514	0.031	(0.028, 0.035)	676	17734	0.035	(0.031, 0.039)	841	71065	0.045	(0.041, 0.050)
Malawi	2015	2005-2009	453	1293	0.026	(0.023, 0.030)	414	15281	0.025	(0.022, 0.028)	570	58648	0.037	(0.033, 0.041)
Malawi	2015	2010-2014	458	1295	0.027	(0.023, 0.030)	256	15372	0.015	(0.013, 0.018)	330	65715	0.020	(0.017, 0.023)
Mali	2012	2002-2006	371	703	0.040	(0.034, 0.045)	294	8290	0.032	(0.027, 0.037)	376	28829	0.049	(0.042, 0.056)
Mali	2012	2007-2011	347	762	0.034	(0.029, 0.039)	205	8805	0.021	(0.018, 0.025)	372	36872	0.039	(0.034, 0.045)
Mali	2018	2008-2012	328	664	0.037	(0.032, 0.043)	273	7870	0.031	(0.027, 0.036)	478	27770	0.065	(0.058, 0.073)
Mali	2018	2013-2017	310	724	0.032	(0.028, 0.037)	210	8453	0.023	(0.019, 0.027)	388	33984	0.044	(0.039, 0.049)

Mozambique	2011	2001-2005	316	701	0.034	(0.029, 0.039)	417	8088	0.046	(0.040, 0.052)	349	29104	0.046	(0.039, 0.052)
Mozambique	2011	2006-2010	338	834	0.031	(0.026, 0.035)	360	9564	0.034	(0.030, 0.039)	302	36669	0.032	(0.027, 0.037)
Namibia	2013	2003-2007	76	327	0.018	(0.013, 0.022)	107	3818	0.025	(0.020, 0.031)	85	14189	0.023	(0.017, 0.029)
Namibia	2013	2008-2012	100	389	0.020	(0.014, 0.025)	92	4462	0.019	(0.014, 0.023)	62	17583	0.014	(0.010, 0.018)
Niger	2012	2002-2006	489	939	0.039	(0.034, 0.044)	511	11152	0.041	(0.037, 0.046)	1083	34928	0.111	(0.102, 0.120)
Niger	2012	2007-2011	302	952	0.024	(0.020, 0.028)	321	10922	0.027	(0.023, 0.030)	873	45275	0.073	(0.067, 0.079)
Nigeria	2013	2003-2007	1301	2227	0.044	(0.040, 0.047)	1248	25937	0.043	(0.040, 0.047)	2064	90181	0.084	(0.078, 0.090)
Nigeria	2013	2008-2012	1198	2393	0.038	(0.035, 0.041)	987	28026	0.032	(0.029, 0.035)	1695	109726	0.059	(0.054, 0.064)
Nigeria	2018	2008-2012	1106	2410	0.035	(0.032, 0.038)	958	28449	0.031	(0.028, 0.033)	1783	99750	0.067	(0.062, 0.072)
Nigeria	2018	2013-2017	1343	2551	0.040	(0.036, 0.043)	942	30252	0.028	(0.026, 0.031)	2069	122448	0.065	(0.060, 0.069)
Rwanda	2010	2000-2004	270	629	0.032	(0.027, 0.037)	344	7328	0.042	(0.037, 0.047)	428	25671	0.062	(0.056, 0.068)
Rwanda	2010	2005-2009	244	684	0.027	(0.023, 0.031)	207	8110	0.023	(0.020, 0.027)	211	33347	0.025	(0.021, 0.028)
Rwanda	2015	2005-2009	198	596	0.025	(0.021, 0.029)	211	7005	0.027	(0.023, 0.031)	221	26162	0.032	(0.028, 0.037)
Rwanda	2015	2010-2014	152	592	0.020	(0.016, 0.023)	97	6967	0.013	(0.010, 0.015)	120	29406	0.016	(0.013, 0.020)
Senegal	2010	2000-2004	389	799	0.037	(0.032, 0.041)	261	9482	0.025	(0.020, 0.030)	429	33640	0.049	(0.043, 0.054)
Senegal	2010	2005-2009	358	932	0.029	(0.025, 0.033)	205	10664	0.017	(0.014, 0.020)	241	42950	0.022	(0.018, 0.026)
Senegal	2012	2002-2006	209	432	0.036	(0.029, 0.043)	112	4987	0.020	(0.016, 0.024)	133	17895	0.029	(0.022, 0.035)
Senegal	2012	2007-2011	175	513	0.026	(0.020, 0.031)	109	5958	0.017	(0.012, 0.022)	124	23235	0.021	(0.016, 0.026)
Senegal	2014	2004-2008	137	440	0.024	(0.018, 0.029)	132	5107	0.023	(0.018, 0.029)	137	18328	0.029	(0.022, 0.035)
Senegal	2014	2009-2013	125	514	0.018	(0.013, 0.024)	96	6023	0.015	(0.011, 0.018)	117	23945	0.019	(0.014, 0.024)
Senegal	2015	2005-2009	182	476	0.029	(0.023, 0.034)	119	5521	0.020	(0.015, 0.025)	152	19508	0.030	(0.023, 0.036)
Senegal	2015	2010-2014	158	526	0.023	(0.018, 0.028)	109	6202	0.016	(0.012, 0.020)	127	25322	0.020	(0.015, 0.024)
Senegal	2016	2006-2010	166	465	0.027	(0.020, 0.034)	104	5470	0.017	(0.014, 0.021)	141	19973	0.027	(0.021, 0.033)
Senegal	2016	2011-2015	138	498	0.021	(0.017, 0.025)	95	5822	0.015	(0.011, 0.018)	83	24473	0.013	(0.010, 0.016)
Senegal	2017	2007-2011	311	868	0.027	(0.023, 0.031)	189	10174	0.017	(0.014, 0.020)	208	37473	0.022	(0.018, 0.025)
Senegal	2017	2012-2016	339	905	0.028	(0.024, 0.032)	153	10695	0.013	(0.011, 0.016)	156	45281	0.014	(0.011, 0.016)
Senegal	2018	2008-2012	175	484	0.027	(0.022, 0.033)	116	5750	0.018	(0.014, 0.022)	122	21117	0.022	(0.018, 0.027)
Senegal	2018	2013-2017	153	498	0.023	(0.019, 0.028)	85	5865	0.013	(0.008, 0.018)	81	25218	0.013	(0.009, 0.016)
Sierra Leone	2013	2003-2007	565	916	0.046	(0.041, 0.052)	997	10624	0.083	(0.075, 0.090)	781	35243	0.080	(0.072, 0.088)
Sierra Leone	2013	2008-2012	457	893	0.038	(0.034, 0.043)	618	10385	0.053	(0.048, 0.059)	667	41999	0.061	(0.055, 0.067)
South Africa	2016	2006-2010	96	254	0.028	(0.018, 0.039)	77	2967	0.024	(0.017, 0.031)	27	11565	0.009	(0.006, 0.012)

South Africa	2016	2011-2015	76	273	0.021	(0.014, 0.029)	51	3237	0.014	(0.009, 0.019)	21	13480	0.006	(0.003, 0.009)
Tanzania	2010	2000-2004	215	544	0.030	(0.024, 0.035)	291	6265	0.042	(0.036, 0.048)	229	22664	0.038	(0.032, 0.045)
Tanzania	2010	2005-2009	214	628	0.026	(0.021, 0.030)	204	7314	0.025	(0.021, 0.030)	214	28641	0.029	(0.024, 0.034)
Tanzania	2015	2005-2009	295	674	0.033	(0.028, 0.038)	253	7863	0.029	(0.024, 0.034)	238	29342	0.031	(0.025, 0.036)
Tanzania	2015	2010-2014	257	767	0.025	(0.021, 0.030)	175	8977	0.018	(0.015, 0.021)	215	35039	0.024	(0.020, 0.028)
Togo	2013	2003-2007	220	516	0.032	(0.026, 0.038)	171	6152	0.025	(0.021, 0.030)	265	22119	0.045	(0.039, 0.051)
Togo	2013	2008-2012	193	538	0.027	(0.023, 0.032)	153	6291	0.022	(0.018, 0.027)	253	25692	0.039	(0.033, 0.044)
Uganda	2011	2001-2005	245	528	0.035	(0.029, 0.041)	299	6083	0.044	(0.038, 0.051)	312	22214	0.053	(0.045, 0.060)
Uganda	2011	2006-2010	210	586	0.027	(0.022, 0.032)	195	6827	0.026	(0.022, 0.030)	239	27289	0.034	(0.028, 0.040)
Uganda	2016	2006-2010	401	1068	0.028	(0.025, 0.032)	355	12449	0.026	(0.023, 0.029)	380	46269	0.031	(0.028, 0.035)
Uganda	2016	2011-2015	411	1163	0.027	(0.024, 0.030)	240	13791	0.016	(0.013, 0.018)	295	56386	0.020	(0.018, 0.023)
Zambia	2013	2003-2007	328	940	0.026	(0.022, 0.030)	326	11052	0.027	(0.023, 0.031)	383	39558	0.037	(0.032, 0.041)
Zambia	2013	2008-2012	325	1017	0.024	(0.021, 0.028)	268	12012	0.020	(0.017, 0.024)	362	49177	0.029	(0.025, 0.032)
Zambia	2018	2008-2012	213	724	0.022	(0.017, 0.027)	192	8554	0.021	(0.017, 0.024)	236	32864	0.028	(0.022, 0.033)
Zambia	2018	2013-2017	274	750	0.028	(0.023, 0.032)	143	8896	0.015	(0.012, 0.017)	156	36958	0.017	(0.013, 0.020)
Zimbabwe	2010	2000-2004	112	338	0.025	(0.019, 0.031)	122	4014	0.028	(0.022, 0.033)	84	15441	0.021	(0.016, 0.026)
Zimbabwe	2010	2005-2009	170	418	0.031	(0.024, 0.038)	136	4665	0.026	(0.022, 0.031)	126	17689	0.027	(0.022, 0.033)
Zimbabwe	2015	2005-2009	168	384	0.033	(0.027, 0.039)	187	4365	0.038	(0.031, 0.046)	138	16235	0.032	(0.025, 0.039)
Zimbabwe	2015	2010-2014	175	462	0.029	(0.023, 0.034)	125	5453	0.021	(0.017, 0.025)	100	21245	0.018	(0.014, 0.022)

Note -- PY: person-years, CI: survey design-based 95% confidence intervals calculated using Taylor linearization.

Table S-3: MICS mortality estimates for Figures 1 and 2.

			Neonatal				Postneonatal				Child			
Country	Survey year	Period	Deaths	PY	q(x)	CI	Deaths	PY	q(x)	CI	Deaths	PY	q(x)	CI
Benin	2014	2004-2008	364	864	0.032	(0.028, 0.036)	385	10059	0.035	(0.030, 0.040)	463	36556	0.048	(0.042, 0.054)
Benin	2014	2009-2013	482	955	0.038	(0.033, 0.043)	361	11131	0.029	(0.025, 0.034)	562	44496	0.049	(0.043, 0.054)
Cameroon	2014	2004-2008	243	513	0.036	(0.030, 0.042)	248	5942	0.038	(0.032, 0.044)	308	20826	0.056	(0.047, 0.064)
Cameroon	2014	2009-2013	207	567	0.028	(0.023, 0.032)	239	6677	0.032	(0.027, 0.037)	286	26305	0.042	(0.036, 0.048)
Congo	2014	2004-2008	138	591	0.018	(0.013, 0.022)	151	6799	0.020	(0.015, 0.026)	105	25404	0.016	(0.013, 0.020)
Congo	2014	2009-2013	192	701	0.021	(0.016, 0.026)	133	8163	0.015	(0.011, 0.018)	137	32828	0.016	(0.013, 0.020)
Cote d'Ivoire	2016	2006-2010	314	621	0.038	(0.033, 0.044)	283	7219	0.035	(0.029, 0.042)	259	25457	0.039	(0.033, 0.045)
Cote d'Ivoire	2016	2011-2015	310	701	0.033	(0.028, 0.038)	238	8184	0.026	(0.022, 0.030)	301	32558	0.036	(0.030, 0.041)
Eswatini	2010	2000-2004	41	162	0.019	(0.013, 0.025)	110	1874	0.053	(0.042, 0.063)	57	7389	0.029	(0.021, 0.037)
Eswatini	2010	2005-2009	47	189	0.019	(0.013, 0.024)	145	2155	0.060	(0.050, 0.070)	51	8329	0.024	(0.017, 0.031)
Eswatini	2014	2004-2008	38	172	0.017	(0.011, 0.022)	129	2001	0.058	(0.036, 0.079)	33	7677	0.017	(0.011, 0.022)
Eswatini	2014	2009-2013	49	184	0.020	(0.013, 0.028)	70	2141	0.030	(0.022, 0.037)	38	8622	0.017	(0.011, 0.024)
Ghana	2011	2001-2005	230	545	0.032	(0.026, 0.038)	135	6358	0.019	(0.015, 0.024)	251	25014	0.039	(0.032, 0.045)
Ghana	2011	2006-2010	248	574	0.033	(0.026, 0.039)	158	6773	0.021	(0.016, 0.026)	201	27881	0.028	(0.023, 0.034)
Guinea	2016	2006-2010	175	550	0.024	(0.020, 0.028)	196	6399	0.028	(0.023, 0.033)	240	22044	0.041	(0.035, 0.048)
Guinea	2016	2011-2015	146	569	0.020	(0.016, 0.023)	168	6833	0.022	(0.018, 0.026)	314	28580	0.043	(0.037, 0.048)
Guinea-Bissau	2014	2004-2008	325	513	0.047	(0.040, 0.055)	173	5941	0.026	(0.021, 0.032)	259	21315	0.046	(0.039, 0.054)
Guinea-Bissau	2014	2009-2013	271	576	0.035	(0.031, 0.040)	147	6804	0.020	(0.016, 0.023)	224	26792	0.033	(0.027, 0.038)
Kenya, Bungoma County	2013	2003-2007	18	69	0.020	(0.008, 0.033)	29	793	0.033	(0.021, 0.045)	18	2908	0.025	(0.011, 0.037)
Kenya, Bungoma County	2013	2008-2012	7	61	0.009	(0.002, 0.015)	7	739	0.009	(0.001, 0.016)	18	3343	0.021	(0.012, 0.030)
Kenya, Kakamega County	2013	2003-2007	17	59	0.022	(0.009, 0.034)	24	686	0.032	(0.017, 0.046)	24	2548	0.035	(0.012, 0.058)
Kenya, Kakamega County	2013	2008-2012	21	58	0.027	(0.014, 0.040)	16	686	0.021	(0.012, 0.031)	16	2936	0.022	(0.007, 0.037)
Kenya, Nyanza Province	2011	2001-2005	122	344	0.027	(0.022, 0.032)	258	3843	0.060	(0.052, 0.068)	180	13855	0.049	(0.042, 0.056)
Kenya, Nyanza Province	2011	2006-2010	130	382	0.026	(0.021, 0.031)	177	4559	0.035	(0.029, 0.041)	143	18156	0.030	(0.025, 0.036)
Kenya, Turkana County	2013	2003-2007	7	67	0.008	(0.002, 0.014)	14	770	0.016	(0.006, 0.026)	12	2734	0.018	(0.007, 0.029)
Kenya, Turkana County	2013	2008-2012	15	79	0.014	(0.005, 0.023)	18	916	0.018	(0.009, 0.027)	22	3693	0.023	(0.013, 0.034)
Madagascar, South	2012	2002-2006	74	185	0.030	(0.021, 0.039)	105	2133	0.044	(0.032, 0.057)	88	7159	0.046	(0.033, 0.059)

Madagascar, South	2012	2007-2011	88	222	0.030	(0.017, 0.042)	89	2519	0.032	(0.024, 0.039)	66	10097	0.025	(0.017, 0.033)
Malawi	2013	2003-2007	575	1420	0.031	(0.027, 0.034)	558	16618	0.031	(0.027, 0.034)	675	60658	0.043	(0.038, 0.047)
Malawi	2013	2008-2012	554	1440	0.029	(0.026, 0.033)	455	17132	0.024	(0.021, 0.027)	547	70482	0.030	(0.027, 0.034)
Mali	2015	2005-2009	554	1181	0.035	(0.031, 0.040)	424	13477	0.029	(0.025, 0.032)	818	46162	0.067	(0.059, 0.074)
Mali	2015	2010-2014	521	1261	0.031	(0.027, 0.035)	409	15134	0.025	(0.021, 0.028)	806	61003	0.051	(0.046, 0.056)
Mauritania	2011	2001-2005	301	668	0.034	(0.029, 0.039)	201	7741	0.024	(0.020, 0.027)	178	27819	0.024	(0.020, 0.028)
Mauritania	2011	2006-2010	314	699	0.034	(0.030, 0.038)	160	8105	0.018	(0.015, 0.021)	135	34498	0.016	(0.013, 0.018)
Mauritania	2015	2005-2009	267	767	0.026	(0.023, 0.030)	126	9004	0.013	(0.010, 0.016)	117	32856	0.014	(0.010, 0.017)
Mauritania	2015	2010-2014	308	814	0.029	(0.025, 0.032)	152	9805	0.014	(0.011, 0.017)	103	41016	0.010	(0.007, 0.012)
Nigeria	2016	2006-2010	979	1987	0.037	(0.034, 0.040)	931	23189	0.036	(0.033, 0.040)	1164	82233	0.053	(0.049, 0.058)
Nigeria	2016	2011-2015	1138	2197	0.039	(0.036, 0.042)	893	25864	0.031	(0.029, 0.034)	1349	103345	0.050	(0.046, 0.054)
Sao Tome and Principe	2014	2004-2008	26	143	0.014	(0.006, 0.021)	19	1684	0.011	(0.004, 0.017)	27	6186	0.017	(0.010, 0.025)
Sao Tome and Principe	2014	2009-2013	44	154	0.022	(0.014, 0.029)	34	1820	0.017	(0.010, 0.024)	11	7645	0.006	(0.002, 0.009)
Senegal	2015	2005-2009	112	320	0.026	(0.020, 0.033)	51	3779	0.012	(0.008, 0.017)	36	14277	0.010	(0.006, 0.014)
Senegal	2015	2010-2014	109	337	0.025	(0.019, 0.030)	34	4055	0.008	(0.005, 0.011)	31	17169	0.007	(0.004, 0.010)
Sierra Leone	2017	2007-2011	272	816	0.025	(0.021, 0.030)	483	9417	0.046	(0.041, 0.051)	428	33281	0.048	(0.042, 0.054)
Sierra Leone	2017	2012-2016	229	876	0.020	(0.017, 0.023)	412	10335	0.036	(0.032, 0.041)	387	41499	0.036	(0.031, 0.041)
Somalia, Northeast Zone	2011	2001-2005	51	384	0.010	(0.007, 0.013)	89	4425	0.018	(0.014, 0.023)	52	15030	0.013	(0.009, 0.017)
Somalia, Northeast Zone	2011	2006-2010	93	353	0.020	(0.015, 0.025)	94	4317	0.020	(0.016, 0.024)	47	19711	0.009	(0.006, 0.013)
Somalia, Somaliland	2011	2001-2005	153	358	0.032	(0.026, 0.038)	173	4099	0.038	(0.032, 0.044)	111	14729	0.029	(0.023, 0.035)
Somalia, Somaliland	2011	2006-2010	200	350	0.043	(0.036, 0.050)	148	4261	0.031	(0.026, 0.037)	81	18244	0.017	(0.013, 0.021)
South Sudan	2010	2000-2004	259	691	0.028	(0.024, 0.033)	236	8000	0.027	(0.022, 0.032)	210	25316	0.031	(0.025, 0.037)
South Sudan	2010	2005-2009	423	736	0.043	(0.037, 0.049)	352	8640	0.037	(0.032, 0.041)	275	35837	0.030	(0.025, 0.034)
Zimbabwe	2014	2004-2008	185	571	0.025	(0.021, 0.029)	245	6512	0.034	(0.029, 0.039)	182	25077	0.028	(0.023, 0.033)
Zimbabwe	2014	2009-2013	276	712	0.029	(0.026, 0.033)	239	8291	0.026	(0.023, 0.030)	154	30842	0.019	(0.015, 0.023)

Note -- PY: person-years, CI: survey design-based 95% confidence intervals calculated using Taylor linearisation.

Table S-4: Data inputs for calculation of differences between HDSS and DHS/MICS subnational region mortality estimates.

Country	Period	Age group	HDSS	Deaths	PY	q(x)	CI	Survey	Year	Region	Deaths	PY	q(x)	CI
Burkina Faso	1998-2002	Neonatal	Nouna	77	602	0.010	(0.008, 0.012)	DHS	2003	Boucle de Mouhoun	17	62	0.021	(0.011, 0.031)
Burkina Faso	1998-2002	Postneonatal	Nouna	393	7278	0.049	(0.044, 0.053)	DHS	2003	Boucle de Mouhoun	34	719	0.042	(0.027, 0.057)
Burkina Faso	1998-2002	Child	Nouna	692	28283	0.091	(0.085, 0.097)	DHS	2003	Boucle de Mouhoun	83	2977	0.105	(0.079, 0.130)
Burkina Faso	2000-2004	Neonatal	Nouna	117	755	0.012	(0.010, 0.014)	DHS	2010	Boucle de Mouhoun	64	108	0.044	(0.033, 0.056)
Burkina Faso	2000-2004	Postneonatal	Nouna	509	9145	0.050	(0.046, 0.054)	DHS	2010	Boucle de Mouhoun	58	1307	0.040	(0.029, 0.051)
Burkina Faso	2000-2004	Child	Nouna	779	36918	0.080	(0.075, 0.085)	DHS	2010	Boucle de Mouhoun	88	4446	0.074	(0.048, 0.098)
Burkina Faso	2005-2009	Neonatal	Nouna	150	999	0.011	(0.010, 0.013)	DHS	2010	Boucle de Mouhoun	34	114	0.022	(0.014, 0.031)
Burkina Faso	2005-2009	Postneonatal	Nouna	423	12003	0.032	(0.029, 0.035)	DHS	2010	Boucle de Mouhoun	47	1315	0.033	(0.017, 0.048)
Burkina Faso	2005-2009	Child	Nouna	856	47030	0.069	(0.065, 0.073)	DHS	2010	Boucle de Mouhoun	89	5264	0.065	(0.046, 0.083)
Cote d'Ivoire	2011-2015	Neonatal	Taabo	113	579	0.015	(0.012, 0.018)	MICS	2016	Sud sans Abidjan	23	58	0.030	(0.015, 0.045)
Cote d'Ivoire	2011-2015	Postneonatal	Taabo	243	7140	0.031	(0.027, 0.035)	MICS	2016	Sud sans Abidjan	20	679	0.027	(0.013, 0.041)
Cote d'Ivoire	2011-2015	Child	Taabo	396	28713	0.052	(0.047, 0.057)	MICS	2016	Sud sans Abidjan	28	2761	0.039	(0.023, 0.054)
Ethiopia	2006-2010	Neonatal	Gilgel Gibe	380	642	0.044	(0.040, 0.049)	DHS	2011	SNNPR	104	229	0.034	(0.025, 0.043)
Ethiopia	2006-2010	Postneonatal	Gilgel Gibe	263	7326	0.032	(0.029, 0.036)	DHS	2011	SNNPR	87	2655	0.030	(0.020, 0.039)
Ethiopia	2006-2010	Child	Gilgel Gibe	274	31013	0.035	(0.031, 0.039)	DHS	2011	SNNPR	92	12104	0.030	(0.021, 0.039)
Ethiopia	2011-2015	Neonatal	Arba Minch	66	752	0.007	(0.005, 0.009)	DHS	2016	SNNPR	63	192	0.025	(0.016, 0.033)
Ethiopia	2011-2015	Postneonatal	Arba Minch	137	8502	0.015	(0.012, 0.017)	DHS	2016	SNNPR	52	2278	0.021	(0.010, 0.031)
Ethiopia	2011-2015	Child	Arba Minch	155	36488	0.017	(0.015, 0.020)	DHS	2016	SNNPR	64	10448	0.025	(0.012, 0.037)
Ethiopia	2011-2015	Neonatal	Dabat	17	441	0.003	(0.002, 0.005)	DHS	2016	Amhara	38	86	0.033	(0.022, 0.044)
Ethiopia	2011-2015	Postneonatal	Dabat	80	6151	0.012	(0.010, 0.015)	DHS	2016	Amhara	16	1015	0.015	(0.007, 0.023)
Ethiopia	2011-2015	Child	Dabat	103	30107	0.014	(0.011, 0.017)	DHS	2016	Amhara	12	4561	0.011	(0.001, 0.021)
Ethiopia	2006-2010	Neonatal	Gilgel Gibe	380	642	0.044	(0.040, 0.049)	DHS	2016	SNNPR	131	220	0.045	(0.031, 0.058)
Ethiopia	2006-2010	Postneonatal	Gilgel Gibe	263	7326	0.032	(0.029, 0.036)	DHS	2016	SNNPR	112	2627	0.038	(0.026, 0.051)
Ethiopia	2006-2010	Child	Gilgel Gibe	274	31013	0.035	(0.031, 0.039)	DHS	2016	SNNPR	58	10104	0.023	(0.012, 0.033)
Ethiopia	2011-2015	Neonatal	Gilgel Gibe	332	713	0.035	(0.032, 0.039)	DHS	2016	SNNPR	63	192	0.025	(0.016, 0.033)
Ethiopia	2011-2015	Postneonatal	Gilgel Gibe	239	8532	0.025	(0.023, 0.029)	DHS	2016	SNNPR	52	2278	0.021	(0.010, 0.031)

Ethiopia	2011-2015	Child	Gilgel Gibe	213	34754	0.024	(0.021, 0.027)	DHS	2016	SNNPR	64	10448	0.025	(0.012, 0.037)
Ethiopia	2011-2015	Neonatal	Kersa	292	821	0.027	(0.024, 0.030)	DHS	2016	Oromia	79	201	0.030	(0.018, 0.041)
Ethiopia	2011-2015	Postneonatal	Kersa	317	10072	0.029	(0.026, 0.032)	DHS	2016	Oromia	55	2340	0.021	(0.013, 0.029)
Ethiopia	2011-2015	Child	Kersa	614	39169	0.061	(0.057, 0.065)	DHS	2016	Oromia	48	10248	0.019	(0.010, 0.027)
Gambia	2003-2007	Neonatal	Farafenni	118	573	0.016	(0.013, 0.019)	DHS	2013	Kerewan	45	80	0.042	(0.025, 0.059)
Gambia	2003-2007	Postneonatal	Farafenni	166	6812	0.022	(0.019, 0.026)	DHS	2013	Kerewan	16	926	0.015	(0.005, 0.025)
Gambia	2003-2007	Child	Farafenni	258	27181	0.037	(0.033, 0.041)	DHS	2013	Kerewan	14	3331	0.017	(0.006, 0.027)
Gambia	2008-2012	Neonatal	Farafenni	98	641	0.012	(0.010, 0.014)	DHS	2013	Kerewan	14	90	0.012	(0.003, 0.020)
Gambia	2008-2012	Postneonatal	Farafenni	104	7713	0.012	(0.010, 0.015)	DHS	2013	Kerewan	2	1040	0.002	(0.000, 0.005)
Gambia	2008-2012	Child	Farafenni	192	32074	0.023	(0.020, 0.027)	DHS	2013	Kerewan	15	4137	0.015	(0.008, 0.022)
Ghana	1993-1997	Neonatal	Navrongo	369	707	0.039	(0.036, 0.043)	DHS	1998	Upper East Region	6	17	0.026	(0.010, 0.042)
Ghana	1993-1997	Postneonatal	Navrongo	720	8209	0.078	(0.073, 0.083)	DHS	1998	Upper East Region	12	200	0.053	(0.027, 0.077)
Ghana	1993-1997	Child	Navrongo	800	33533	0.091	(0.086, 0.097)	DHS	1998	Upper East Region	15	890	0.064	(0.034, 0.093)
Ghana	1993-1997	Neonatal	Navrongo	369	707	0.039	(0.036, 0.043)	DHS	2003	Upper East Region	3	18	0.013	(0.002, 0.024)
Ghana	1993-1997	Postneonatal	Navrongo	720	8209	0.078	(0.073, 0.083)	DHS	2003	Upper East Region	5	231	0.020	(0.003, 0.036)
Ghana	1993-1997	Child	Navrongo	800	33533	0.091	(0.086, 0.097)	DHS	2003	Upper East Region	14	855	0.063	(0.030, 0.094)
Ghana	1998-2002	Neonatal	Navrongo	332	776	0.032	(0.029, 0.036)	DHS	2003	Upper East Region	7	17	0.031	(0.006, 0.056)
Ghana	1998-2002	Postneonatal	Navrongo	543	9191	0.053	(0.049, 0.057)	DHS	2003	Upper East Region	1	201	0.003	(0.000, 0.010)
Ghana	1998-2002	Child	Navrongo	644	37274	0.066	(0.062, 0.071)	DHS	2003	Upper East Region	7	883	0.030	(0.008, 0.052)
Ghana	1998-2002	Neonatal	Navrongo	332	776	0.032	(0.029, 0.036)	DHS	2008	Upper East Region	3	13	0.020	(0.002, 0.038)
Ghana	1998-2002	Postneonatal	Navrongo	543	9191	0.053	(0.049, 0.057)	DHS	2008	Upper East Region	7	151	0.041	(0.008, 0.072)
Ghana	1998-2002	Child	Navrongo	644	37274	0.066	(0.062, 0.071)	DHS	2008	Upper East Region	5	598	0.031	(0.011, 0.050)
Ghana	2003-2007	Neonatal	Navrongo	236	757	0.024	(0.021, 0.027)	DHS	2008	Upper East Region	2	11	0.013	(0.000, 0.027)
Ghana	2003-2007	Postneonatal	Navrongo	345	8870	0.035	(0.032, 0.039)	DHS	2008	Upper East Region	3	133	0.020	(0.000, 0.039)
Ghana	2003-2007	Child	Navrongo	433	36472	0.046	(0.043, 0.051)	DHS	2008	Upper East Region	4	598	0.023	(0.004, 0.042)
Ghana	2006-2010	Neonatal	Dodowa	85	948	0.007	(0.006, 0.008)	MICS	2011	Greater Accra Region	22	76	0.022	(0.009, 0.035)
Ghana	2006-2010	Postneonatal	Dodowa	112	12008	0.009	(0.007, 0.010)	MICS	2011	Greater Accra Region	20	872	0.021	(0.002, 0.040)
Ghana	2006-2010	Child	Dodowa	277	57704	0.019	(0.017, 0.021)	MICS	2011	Greater Accra Region	16	3575	0.017	(0.002, 0.032)
Ghana	2006-2010	Neonatal	Kintampo	273	1402	0.015	(0.013, 0.017)	MICS	2011	Brong Ahafo	35	46	0.056	(0.027, 0.085)
Ghana	2006-2010	Postneonatal	Kintampo	335	16889	0.018	(0.016, 0.020)	MICS	2011	Brong Ahafo	17	544	0.028	(0.008, 0.047)

Ghana	2006-2010	Child	Kintampo	493	66596	0.029	(0.027, 0.031)	MICS	2011	Brong Ahafo	13	2327	0.023	(0.009, 0.036)
Ghana	2001-2005	Neonatal	Navrongo	278	759	0.028	(0.025, 0.031)	MICS	2011	Upper East Region	11	25	0.034	(0.023, 0.046)
Ghana	2001-2005	Postneonatal	Navrongo	413	9059	0.041	(0.038, 0.045)	MICS	2011	Upper East Region	10	299	0.032	(0.019, 0.044)
Ghana	2001-2005	Child	Navrongo	510	37052	0.053	(0.049, 0.058)	MICS	2011	Upper East Region	15	1161	0.051	(0.034, 0.067)
Ghana	2006-2010	Neonatal	Navrongo	188	751	0.019	(0.017, 0.022)	MICS	2011	Upper East Region	10	22	0.034	(0.019, 0.048)
Ghana	2006-2010	Postneonatal	Navrongo	219	8854	0.023	(0.020, 0.026)	MICS	2011	Upper East Region	5	268	0.016	(0.006, 0.027)
Ghana	2006-2010	Child	Navrongo	322	36715	0.034	(0.031, 0.038)	MICS	2011	Upper East Region	9	1202	0.030	(0.018, 0.042)
Ghana	2009-2013	Neonatal	Kintampo	433	1660	0.020	(0.018, 0.022)	DHS	2014	Brong Ahafo	15	40	0.028	(0.015, 0.040)
Ghana	2009-2013	Postneonatal	Kintampo	331	20390	0.015	(0.013, 0.017)	DHS	2014	Brong Ahafo	3	478	0.006	(0.000, 0.013)
Ghana	2009-2013	Child	Kintampo	516	81359	0.025	(0.023, 0.027)	DHS	2014	Brong Ahafo	7	1862	0.014	(0.006, 0.022)
Ghana	2004-2008	Neonatal	Navrongo	230	739	0.024	(0.021, 0.027)	DHS	2014	Upper East Region	6	18	0.025	(0.010, 0.040)
Ghana	2004-2008	Postneonatal	Navrongo	299	8833	0.031	(0.028, 0.034)	DHS	2014	Upper East Region	8	209	0.034	(0.015, 0.052)
Ghana	2004-2008	Child	Navrongo	388	36412	0.042	(0.038, 0.046)	DHS	2014	Upper East Region	8	790	0.039	(0.024, 0.053)
Ghana	2009-2013	Neonatal	Navrongo	133	775	0.013	(0.011, 0.015)	DHS	2014	Upper East Region	6	18	0.023	(0.007, 0.039)
Ghana	2009-2013	Postneonatal	Navrongo	155	9246	0.015	(0.013, 0.018)	DHS	2014	Upper East Region	2	213	0.011	(0.001, 0.020)
Ghana	2009-2013	Child	Navrongo	255	38195	0.026	(0.023, 0.030)	DHS	2014	Upper East Region	4	867	0.016	(0.006, 0.027)
Kenya	2003-2007	Neonatal	Nairobi	128	714	0.014	(0.012, 0.016)	DHS	2008	Nairobi	18	25	0.054	(0.016, 0.090)
Kenya	2003-2007	Postneonatal	Nairobi	357	8745	0.037	(0.034, 0.041)	DHS	2008	Nairobi	3	276	0.010	(0.000, 0.022)
Kenya	2003-2007	Child	Nairobi	248	31164	0.030	(0.026, 0.033)	DHS	2008	Nairobi	1	1071	0.005	(0.000, 0.011)
Kenya	2009-2013	Neonatal	Mbita	38	374	0.008	(0.006, 0.011)	DHS	2014	Nyanza	100	386	0.020	(0.014, 0.026)
Kenya	2009-2013	Postneonatal	Mbita	177	5045	0.032	(0.028, 0.037)	DHS	2014	Nyanza	125	4599	0.025	(0.018, 0.031)
Kenya	2009-2013	Child	Mbita	234	23699	0.039	(0.034, 0.044)	DHS	2014	Nyanza	117	20465	0.023	(0.017, 0.029)
Kenya	2004-2008	Neonatal	Nairobi	130	722	0.014	(0.012, 0.016)	DHS	2014	Nairobi	60	103	0.043	(0.021, 0.065)
Kenya	2004-2008	Postneonatal	Nairobi	343	8815	0.035	(0.032, 0.039)	DHS	2014	Nairobi	15	1223	0.011	(0.000, 0.025)
Kenya	2004-2008	Child	Nairobi	236	31815	0.028	(0.024, 0.031)	DHS	2014	Nairobi	17	4518	0.015	(0.002, 0.028)
Kenya	2009-2013	Neonatal	Nairobi	220	744	0.022	(0.020, 0.025)	DHS	2014	Nairobi	68	139	0.037	(0.016, 0.057)
Kenya	2009-2013	Postneonatal	Nairobi	324	9084	0.032	(0.029, 0.036)	DHS	2014	Nairobi	36	1618	0.020	(0.009, 0.032)
Kenya	2009-2013	Child	Nairobi	192	34759	0.021	(0.018, 0.024)	DHS	2014	Nairobi	26	6120	0.017	(0.005, 0.028)
Malawi	2005-2009	Neonatal	Karonga	158	529	0.023	(0.019, 0.026)	DHS	2010	Northern Region	157	269	0.044	(0.030, 0.057)
Malawi	2005-2009	Postneonatal	Karonga	164	6243	0.024	(0.021, 0.028)	DHS	2010	Northern Region	99	3113	0.029	(0.019, 0.039)

Malawi	2005-2009	Child	Karonga	197	25251	0.030	(0.026, 0.034)	DHS	2010	Northern Region	89	12521	0.027	(0.018, 0.037)
Malawi	2003-2007	Neonatal	Karonga	140	475	0.022	(0.019, 0.026)	MICS	2013	Northern Region	19	53	0.027	(0.012, 0.041)
Malawi	2003-2007	Postneonatal	Karonga	161	5594	0.026	(0.023, 0.030)	MICS	2013	Northern Region	18	607	0.026	(0.014, 0.038)
Malawi	2003-2007	Child	Karonga	193	21374	0.034	(0.030, 0.039)	MICS	2013	Northern Region	15	2241	0.025	(0.012, 0.039)
Malawi	2008-2012	Neonatal	Karonga	131	503	0.020	(0.017, 0.023)	MICS	2013	Northern Region	12	48	0.019	(0.007, 0.030)
Malawi	2008-2012	Postneonatal	Karonga	129	6001	0.020	(0.017, 0.023)	MICS	2013	Northern Region	12	575	0.019	(0.008, 0.030)
Malawi	2008-2012	Child	Karonga	174	25448	0.027	(0.023, 0.031)	MICS	2013	Northern Region	11	2525	0.018	(0.007, 0.029)
Malawi	2005-2009	Neonatal	Karonga	158	529	0.023	(0.019, 0.026)	DHS	2015	Northern Region	76	254	0.023	(0.015, 0.030)
Malawi	2005-2009	Postneonatal	Karonga	164	6243	0.024	(0.021, 0.028)	DHS	2015	Northern Region	68	3019	0.021	(0.013, 0.028)
Malawi	2005-2009	Child	Karonga	197	25251	0.030	(0.026, 0.034)	DHS	2015	Northern Region	77	11740	0.025	(0.018, 0.033)
Malawi	2010-2014	Neonatal	Karonga	144	494	0.022	(0.019, 0.026)	DHS	2015	Northern Region	62	244	0.019	(0.013, 0.026)
Malawi	2010-2014	Postneonatal	Karonga	97	5921	0.015	(0.012, 0.018)	DHS	2015	Northern Region	36	2915	0.011	(0.006, 0.017)
Malawi	2010-2014	Child	Karonga	137	25242	0.021	(0.018, 0.025)	DHS	2015	Northern Region	46	12691	0.014	(0.009, 0.020)
Nigeria	2013-2017	Neonatal	Cross River	4	16	0.019	(0.007, 0.049)	DHS	2018	South south	72	197	0.028	(0.020, 0.035)
Nigeria	2013-2017	Postneonatal	Cross River	12	1326	0.009	(0.005, 0.016)	DHS	2018	South south	59	2339	0.023	(0.018, 0.029)
Nigeria	2013-2017	Child	Cross River	36	11849	0.013	(0.009, 0.018)	DHS	2018	South south	61	9267	0.026	(0.016, 0.035)
Senegal	1992-1996	Neonatal	Bandafassi	119	148	0.060	(0.051, 0.070)	DHS	1997	Kedougou	83	115	0.054	(0.040, 0.068)
Senegal	1992-1996	Postneonatal	Bandafassi	158	1670	0.083	(0.072, 0.095)	DHS	1997	Kedougou	53	1321	0.037	(0.025, 0.048)
Senegal	1992-1996	Child	Bandafassi	219	5929	0.133	(0.120, 0.148)	DHS	1997	Kedougou	105	5463	0.073	(0.059, 0.087)
Senegal	1992-1996	Neonatal	Mlomp	21	66	0.024	(0.016, 0.036)	DHS	1997	Ziguinchor	54	93	0.043	(0.028, 0.058)
Senegal	1992-1996	Postneonatal	Mlomp	34	761	0.040	(0.029, 0.055)	DHS	1997	Ziguinchor	51	1079	0.043	(0.027, 0.057)
Senegal	1992-1996	Child	Mlomp	50	3347	0.058	(0.045, 0.075)	DHS	1997	Ziguinchor	110	4250	0.097	(0.077, 0.116)
Senegal	1992-1996	Neonatal	Niakhar	209	421	0.037	(0.033, 0.042)	DHS	1997	Fatick	118	253	0.035	(0.028, 0.043)
Senegal	1992-1996	Postneonatal	Niakhar	263	4907	0.048	(0.043, 0.054)	DHS	1997	Fatick	98	2918	0.031	(0.025, 0.036)
Senegal	1992-1996	Child	Niakhar	663	18725	0.130	(0.122, 0.138)	DHS	1997	Fatick	258	11592	0.084	(0.072, 0.096)
Senegal	1995-1999	Neonatal	Bandafassi	124	156	0.059	(0.050, 0.069)	DHS	2005	Kedougou	84	82	0.075	(0.054, 0.096)
Senegal	1995-1999	Postneonatal	Bandafassi	153	1758	0.077	(0.067, 0.089)	DHS	2005	Kedougou	61	929	0.059	(0.039, 0.078)
Senegal	1995-1999	Child	Bandafassi	218	6335	0.124	(0.111, 0.138)	DHS	2005	Kedougou	114	3064	0.135	(0.094, 0.174)
Senegal	2000-2004	Neonatal	Bandafassi	121	193	0.047	(0.040, 0.055)	DHS	2005	Kedougou	46	88	0.040	(0.026, 0.053)
Senegal	2000-2004	Postneonatal	Bandafassi	137	2180	0.056	(0.048, 0.066)	DHS	2005	Kedougou	38	1011	0.034	(0.022, 0.046)

Senegal	2000-2004	Child	Bandafassi	219	7609	0.104	(0.093, 0.117)	DHS	2005	Kedougou	90	3875	0.087	(0.065, 0.109)
Senegal	1995-1999	Neonatal	Mlomp	26	62	0.032	(0.022, 0.045)	DHS	2005	Ziguinchor	23	39	0.044	(0.022, 0.066)
Senegal	1995-1999	Postneonatal	Mlomp	17	729	0.021	(0.013, 0.034)	DHS	2005	Ziguinchor	22	457	0.043	(0.024, 0.061)
Senegal	1995-1999	Child	Mlomp	46	3250	0.055	(0.042, 0.072)	DHS	2005	Ziguinchor	28	1649	0.063	(0.040, 0.086)
Senegal	2000-2004	Neonatal	Mlomp	19	68	0.021	(0.014, 0.033)	DHS	2005	Ziguinchor	11	41	0.020	(0.010, 0.030)
Senegal	2000-2004	Postneonatal	Mlomp	17	754	0.020	(0.013, 0.032)	DHS	2005	Ziguinchor	19	469	0.036	(0.022, 0.050)
Senegal	2000-2004	Child	Mlomp	42	3287	0.050	(0.038, 0.066)	DHS	2005	Ziguinchor	28	1870	0.059	(0.043, 0.074)
Senegal	1995-1999	Neonatal	Niakhar	165	435	0.029	(0.025, 0.033)	DHS	2005	Fatick	65	61	0.079	(0.055, 0.102)
Senegal	1995-1999	Postneonatal	Niakhar	330	5003	0.059	(0.054, 0.065)	DHS	2005	Fatick	19	720	0.024	(0.013, 0.036)
Senegal	1995-1999	Child	Niakhar	849	19030	0.161	(0.153, 0.169)	DHS	2005	Fatick	71	2677	0.098	(0.077, 0.119)
Senegal	2000-2004	Neonatal	Niakhar	151	484	0.024	(0.020, 0.028)	DHS	2005	Fatick	36	70	0.039	(0.021, 0.056)
Senegal	2000-2004	Postneonatal	Niakhar	263	5627	0.042	(0.038, 0.047)	DHS	2005	Fatick	22	790	0.025	(0.017, 0.033)
Senegal	2000-2004	Child	Niakhar	469	20286	0.085	(0.079, 0.092)	DHS	2005	Fatick	44	3001	0.056	(0.028, 0.084)
Senegal	2000-2004	Neonatal	Bandafassi	121	193	0.047	(0.040, 0.055)	DHS	2010	Kedougou	7	15	0.035	(0.010, 0.059)
Senegal	2000-2004	Postneonatal	Bandafassi	137	2180	0.056	(0.048, 0.066)	DHS	2010	Kedougou	8	172	0.042	(0.022, 0.062)
Senegal	2000-2004	Child	Bandafassi	219	7609	0.104	(0.093, 0.117)	DHS	2010	Kedougou	18	544	0.121	(0.075, 0.164)
Senegal	2005-2009	Neonatal	Bandafassi	78	193	0.031	(0.025, 0.038)	DHS	2010	Kedougou	5	14	0.024	(0.009, 0.039)
Senegal	2005-2009	Postneonatal	Bandafassi	108	2322	0.042	(0.035, 0.050)	DHS	2010	Kedougou	6	162	0.033	(0.015, 0.051)
Senegal	2005-2009	Child	Bandafassi	213	8731	0.091	(0.081, 0.102)	DHS	2010	Kedougou	11	704	0.061	(0.038, 0.083)
Senegal	2000-2004	Neonatal	Mlomp	19	68	0.021	(0.014, 0.033)	DHS	2010	Ziguinchor	13	40	0.025	(0.011, 0.038)
Senegal	2000-2004	Postneonatal	Mlomp	17	754	0.020	(0.013, 0.032)	DHS	2010	Ziguinchor	21	459	0.040	(0.021, 0.059)
Senegal	2000-2004	Child	Mlomp	42	3287	0.050	(0.038, 0.066)	DHS	2010	Ziguinchor	10	1714	0.023	(0.010, 0.036)
Senegal	2005-2009	Neonatal	Mlomp	24	68	0.027	(0.018, 0.039)	DHS	2010	Ziguinchor	18	48	0.029	(0.013, 0.044)
Senegal	2005-2009	Postneonatal	Mlomp	12	762	0.014	(0.008, 0.024)	DHS	2010	Ziguinchor	10	557	0.016	(0.001, 0.030)
Senegal	2005-2009	Child	Mlomp	16	3163	0.020	(0.013, 0.033)	DHS	2010	Ziguinchor	9	2182	0.016	(0.005, 0.027)
Senegal	2000-2004	Neonatal	Niakhar	151	484	0.024	(0.020, 0.028)	DHS	2010	Fatick	28	75	0.028	(0.016, 0.040)
Senegal	2000-2004	Postneonatal	Niakhar	263	5627	0.042	(0.038, 0.047)	DHS	2010	Fatick	21	891	0.022	(0.011, 0.032)
Senegal	2000-2004	Child	Niakhar	469	20286	0.085	(0.079, 0.092)	DHS	2010	Fatick	55	3158	0.066	(0.043, 0.088)
Senegal	2005-2009	Neonatal	Niakhar	75	550	0.010	(0.008, 0.013)	DHS	2010	Fatick	28	86	0.025	(0.012, 0.038)
Senegal	2005-2009	Postneonatal	Niakhar	136	6556	0.019	(0.016, 0.022)	DHS	2010	Fatick	24	971	0.022	(0.010, 0.034)

Senegal	2005-2009	Child	Niakhar	310	25115	0.047	(0.042, 0.052)	DHS	2010	Fatick	18	3855	0.018	(0.006, 0.030)
Senegal	2002-2006	Neonatal	Bandafassi	119	195	0.046	(0.039, 0.054)	DHS	2012	Kedougou	7	9	0.052	(0.027, 0.076)
Senegal	2002-2006	Postneonatal	Bandafassi	105	2230	0.043	(0.036, 0.051)	DHS	2012	Kedougou	6	105	0.054	(0.019, 0.089)
Senegal	2002-2006	Child	Bandafassi	181	8162	0.082	(0.072, 0.093)	DHS	2012	Kedougou	9	330	0.096	(0.075, 0.117)
Senegal	2007-2011	Neonatal	Bandafassi	55	192	0.022	(0.017, 0.028)	DHS	2012	Kedougou	3	11	0.019	(0.001, 0.036)
Senegal	2007-2011	Postneonatal	Bandafassi	101	2266	0.040	(0.033, 0.048)	DHS	2012	Kedougou	2	127	0.017	(0.000, 0.035)
Senegal	2007-2011	Child	Bandafassi	180	8993	0.076	(0.067, 0.086)	DHS	2012	Kedougou	4	480	0.035	(0.000, 0.076)
Senegal	2002-2006	Neonatal	Mlomp	16	67	0.018	(0.011, 0.029)	DHS	2012	Ziguinchor	6	18	0.025	(0.000, 0.050)
Senegal	2002-2006	Postneonatal	Mlomp	17	743	0.020	(0.013, 0.032)	DHS	2012	Ziguinchor	1	217	0.005	(0.000, 0.014)
Senegal	2002-2006	Child	Mlomp	23	3317	0.028	(0.019, 0.041)	DHS	2012	Ziguinchor	3	764	0.015	(0.000, 0.042)
Senegal	2007-2011	Neonatal	Mlomp	23	67	0.026	(0.018, 0.038)	DHS	2012	Ziguinchor	9	24	0.029	(0.000, 0.057)
Senegal	2007-2011	Postneonatal	Mlomp	9	780	0.011	(0.006, 0.020)	DHS	2012	Ziguinchor	3	261	0.011	(0.000, 0.028)
Senegal	2007-2011	Child	Mlomp	12	3238	0.015	(0.009, 0.026)	DHS	2012	Ziguinchor	1	1000	0.005	(0.000, 0.014)
Senegal	2002-2006	Neonatal	Niakhar	128	509	0.019	(0.016, 0.023)	DHS	2012	Fatick	13	34	0.029	(0.012, 0.047)
Senegal	2002-2006	Postneonatal	Niakhar	202	5932	0.031	(0.027, 0.035)	DHS	2012	Fatick	14	383	0.032	(0.015, 0.048)
Senegal	2002-2006	Child	Niakhar	419	22315	0.070	(0.064, 0.076)	DHS	2012	Fatick	10	1410	0.029	(0.006, 0.052)
Senegal	2007-2011	Neonatal	Niakhar	59	568	0.008	(0.006, 0.010)	DHS	2012	Fatick	11	43	0.020	(0.006, 0.033)
Senegal	2007-2011	Postneonatal	Niakhar	109	6944	0.014	(0.012, 0.017)	DHS	2012	Fatick	5	500	0.008	(0.000, 0.020)
Senegal	2007-2011	Child	Niakhar	219	27136	0.031	(0.027, 0.035)	DHS	2012	Fatick	15	1906	0.031	(0.006, 0.055)
Senegal	2004-2008	Neonatal	Bandafassi	92	199	0.035	(0.029, 0.042)	DHS	2014	Kedougou	4	10	0.031	(0.002, 0.059)
Senegal	2004-2008	Postneonatal	Bandafassi	100	2277	0.040	(0.033, 0.048)	DHS	2014	Kedougou	4	126	0.031	(0.008, 0.054)
Senegal	2004-2008	Child	Bandafassi	212	8604	0.092	(0.081, 0.103)	DHS	2014	Kedougou	6	438	0.051	(0.014, 0.087)
Senegal	2009-2013	Neonatal	Bandafassi	51	186	0.021	(0.016, 0.027)	DHS	2014	Kedougou	6	12	0.038	(0.006, 0.068)
Senegal	2009-2013	Postneonatal	Bandafassi	81	2248	0.033	(0.027, 0.040)	DHS	2014	Kedougou	1	140	0.005	(0.000, 0.013)
Senegal	2009-2013	Child	Bandafassi	133	9023	0.057	(0.049, 0.066)	DHS	2014	Kedougou	8	532	0.059	(0.027, 0.090)
Senegal	2004-2008	Neonatal	Mlomp	20	67	0.023	(0.015, 0.034)	DHS	2014	Ziguinchor	1	24	0.004	(0.000, 0.011)
Senegal	2004-2008	Postneonatal	Mlomp	13	758	0.015	(0.009, 0.026)	DHS	2014	Ziguinchor	9	288	0.028	(0.004, 0.052)
Senegal	2004-2008	Child	Mlomp	16	3160	0.020	(0.013, 0.033)	DHS	2014	Ziguinchor	5	1053	0.018	(0.000, 0.040)
Senegal	2009-2013	Neonatal	Mlomp	24	71	0.025	(0.017, 0.037)	DHS	2014	Ziguinchor	5	29	0.013	(0.002, 0.024)
Senegal	2009-2013	Postneonatal	Mlomp	9	811	0.010	(0.005, 0.019)	DHS	2014	Ziguinchor	8	340	0.020	(0.004, 0.036)

Senegal	2009-2013	Child	Mlomp	7	3391	0.008	(0.004, 0.017)	DHS	2014	Ziguinchor	5	1321	0.014	(0.002, 0.026)
Senegal	2004-2008	Neonatal	Niakhar	88	535	0.013	(0.010, 0.015)	DHS	2014	Fatick	13	36	0.028	(0.002, 0.054)
Senegal	2004-2008	Postneonatal	Niakhar	147	6313	0.021	(0.018, 0.025)	DHS	2014	Fatick	11	426	0.025	(0.010, 0.039)
Senegal	2004-2008	Child	Niakhar	349	24212	0.055	(0.050, 0.060)	DHS	2014	Fatick	11	1564	0.027	(0.006, 0.047)
Senegal	2009-2013	Neonatal	Niakhar	85	597	0.011	(0.009, 0.013)	DHS	2014	Fatick	11	43	0.019	(0.003, 0.035)
Senegal	2009-2013	Postneonatal	Niakhar	106	7274	0.013	(0.011, 0.016)	DHS	2014	Fatick	3	500	0.005	(0.000, 0.012)
Senegal	2009-2013	Child	Niakhar	205	29084	0.027	(0.024, 0.031)	DHS	2014	Fatick	5	1959	0.009	(0.000, 0.018)
Senegal	2005-2009	Neonatal	Bandafassi	78	193	0.031	(0.025, 0.038)	DHS	2015	Kedougou	4	7	0.041	(0.008, 0.073)
Senegal	2005-2009	Postneonatal	Bandafassi	108	2322	0.042	(0.035, 0.050)	DHS	2015	Kedougou	5	86	0.050	(0.028, 0.071)
Senegal	2005-2009	Child	Bandafassi	213	8731	0.091	(0.081, 0.102)	DHS	2015	Kedougou	6	299	0.080	(0.037, 0.121)
Senegal	2010-2014	Neonatal	Bandafassi	62	195	0.024	(0.019, 0.031)	DHS	2015	Kedougou	4	10	0.033	(0.016, 0.050)
Senegal	2010-2014	Postneonatal	Bandafassi	69	2193	0.028	(0.023, 0.036)	DHS	2015	Kedougou	5	117	0.040	(0.016, 0.063)
Senegal	2010-2014	Child	Bandafassi	120	9043	0.051	(0.043, 0.060)	DHS	2015	Kedougou	5	417	0.051	(0.014, 0.086)
Senegal	2005-2009	Neonatal	Mlomp	24	68	0.027	(0.018, 0.039)	DHS	2015	Ziguinchor	20	27	0.055	(0.025, 0.084)
Senegal	2005-2009	Postneonatal	Mlomp	12	762	0.014	(0.008, 0.024)	DHS	2015	Ziguinchor	11	314	0.031	(0.010, 0.051)
Senegal	2005-2009	Child	Mlomp	16	3163	0.020	(0.013, 0.033)	DHS	2015	Ziguinchor	7	1111	0.023	(0.004, 0.041)
Senegal	2010-2014	Neonatal	Mlomp	20	70	0.022	(0.014, 0.033)	DHS	2015	Ziguinchor	10	28	0.027	(0.004, 0.050)
Senegal	2010-2014	Postneonatal	Mlomp	8	827	0.009	(0.004, 0.018)	DHS	2015	Ziguinchor	6	332	0.016	(0.003, 0.028)
Senegal	2010-2014	Child	Mlomp	8	3478	0.009	(0.005, 0.018)	DHS	2015	Ziguinchor	2	1385	0.007	(0.000, 0.019)
Senegal	2005-2009	Neonatal	Niakhar	75	550	0.010	(0.008, 0.013)	DHS	2015	Fatick	10	42	0.018	(0.004, 0.033)
Senegal	2005-2009	Postneonatal	Niakhar	136	6556	0.019	(0.016, 0.022)	DHS	2015	Fatick	8	490	0.016	(0.004, 0.027)
Senegal	2005-2009	Child	Niakhar	310	25115	0.047	(0.042, 0.052)	DHS	2015	Fatick	17	1738	0.037	(0.015, 0.059)
Senegal	2010-2014	Neonatal	Niakhar	92	603	0.012	(0.010, 0.014)	DHS	2015	Fatick	24	47	0.038	(0.013, 0.063)
Senegal	2010-2014	Postneonatal	Niakhar	107	7356	0.013	(0.011, 0.016)	DHS	2015	Fatick	6	553	0.010	(0.000, 0.023)
Senegal	2010-2014	Child	Niakhar	209	29849	0.027	(0.024, 0.031)	DHS	2015	Fatick	13	2197	0.023	(0.007, 0.038)
Senegal	2006-2010	Neonatal	Bandafassi	63	195	0.024	(0.019, 0.031)	DHS	2016	Kedougou	7	13	0.042	(0.022, 0.062)
Senegal	2006-2010	Postneonatal	Bandafassi	105	2267	0.042	(0.035, 0.050)	DHS	2016	Kedougou	8	142	0.050	(0.028, 0.071)
Senegal	2006-2010	Child	Bandafassi	191	8893	0.081	(0.071, 0.092)	DHS	2016	Kedougou	16	484	0.120	(0.090, 0.149)
Senegal	2011-2015	Neonatal	Bandafassi	69	193	0.027	(0.022, 0.034)	DHS	2016	Kedougou	5	14	0.024	(0.009, 0.038)
Senegal	2011-2015	Postneonatal	Bandafassi	70	2280	0.028	(0.022, 0.035)	DHS	2016	Kedougou	4	167	0.023	(0.008, 0.037)

Senegal	2011-2015	Child	Bandafassi	120	9073	0.051	(0.043, 0.060)	DHS	2016	Kedougou	8	642	0.048	(0.021, 0.074)
Senegal	2006-2010	Neonatal	Mlomp	23	68	0.026	(0.017, 0.038)	DHS	2016	Ziguinchor	6	22	0.020	(0.003, 0.037)
Senegal	2006-2010	Postneonatal	Mlomp	10	769	0.012	(0.006, 0.022)	DHS	2016	Ziguinchor	7	260	0.025	(0.000, 0.052)
Senegal	2006-2010	Child	Mlomp	13	3173	0.017	(0.010, 0.028)	DHS	2016	Ziguinchor	4	1020	0.016	(0.000, 0.046)
Senegal	2011-2015	Neonatal	Mlomp	18	70	0.019	(0.012, 0.030)	DHS	2016	Ziguinchor	9	22	0.030	(0.009, 0.051)
Senegal	2011-2015	Postneonatal	Mlomp	7	810	0.008	(0.004, 0.016)	DHS	2016	Ziguinchor	2	260	0.006	(0.000, 0.017)
Senegal	2011-2015	Child	Mlomp	9	3536	0.010	(0.005, 0.019)	DHS	2016	Ziguinchor	0	1135	0.000	(0.000, 0.000)
Senegal	2006-2010	Neonatal	Niakhar	68	567	0.009	(0.007, 0.012)	DHS	2016	Fatick	14	49	0.022	(0.007, 0.038)
Senegal	2006-2010	Postneonatal	Niakhar	117	6771	0.016	(0.013, 0.019)	DHS	2016	Fatick	11	576	0.017	(0.005, 0.028)
Senegal	2006-2010	Child	Niakhar	263	26094	0.038	(0.034, 0.043)	DHS	2016	Fatick	13	2143	0.022	(0.008, 0.037)
Senegal	2011-2015	Neonatal	Niakhar	101	596	0.013	(0.011, 0.016)	DHS	2016	Fatick	15	50	0.023	(0.008, 0.038)
Senegal	2011-2015	Postneonatal	Niakhar	100	7374	0.012	(0.010, 0.015)	DHS	2016	Fatick	5	591	0.008	(0.001, 0.015)
Senegal	2011-2015	Child	Niakhar	187	30409	0.024	(0.021, 0.027)	DHS	2016	Fatick	8	2472	0.013	(0.002, 0.025)
Senegal	2007-2011	Neonatal	Bandafassi	55	192	0.022	(0.017, 0.028)	DHS	2017	Kedougou	5	17	0.023	(0.012, 0.034)
Senegal	2007-2011	Postneonatal	Bandafassi	101	2266	0.040	(0.033, 0.048)	DHS	2017	Kedougou	5	206	0.020	(0.004, 0.036)
Senegal	2007-2011	Child	Bandafassi	180	8993	0.076	(0.067, 0.086)	DHS	2017	Kedougou	8	719	0.042	(0.018, 0.066)
Senegal	2012-2016	Neonatal	Bandafassi	63	190	0.025	(0.020, 0.032)	DHS	2017	Kedougou	7	21	0.025	(0.011, 0.039)
Senegal	2012-2016	Postneonatal	Bandafassi	62	2249	0.025	(0.020, 0.032)	DHS	2017	Kedougou	3	237	0.011	(0.003, 0.019)
Senegal	2012-2016	Child	Bandafassi	97	9185	0.041	(0.034, 0.049)	DHS	2017	Kedougou	6	940	0.024	(0.010, 0.037)
Senegal	2007-2011	Neonatal	Mlomp	23	67	0.026	(0.018, 0.038)	DHS	2017	Ziguinchor	17	51	0.025	(0.006, 0.043)
Senegal	2007-2011	Postneonatal	Mlomp	9	780	0.011	(0.006, 0.020)	DHS	2017	Ziguinchor	13	591	0.021	(0.005, 0.036)
Senegal	2007-2011	Child	Mlomp	12	3238	0.015	(0.009, 0.026)	DHS	2017	Ziguinchor	6	2209	0.011	(0.003, 0.019)
Senegal	2012-2016	Neonatal	Mlomp	13	62	0.016	(0.009, 0.027)	DHS	2017	Ziguinchor	20	56	0.026	(0.012, 0.040)
Senegal	2012-2016	Postneonatal	Mlomp	5	779	0.006	(0.002, 0.014)	DHS	2017	Ziguinchor	10	649	0.014	(0.005, 0.023)
Senegal	2012-2016	Child	Mlomp	6	3516	0.007	(0.003, 0.015)	DHS	2017	Ziguinchor	9	2670	0.013	(0.005, 0.021)
Senegal	2007-2011	Neonatal	Niakhar	59	568	0.008	(0.006, 0.010)	DHS	2017	Fatick	30	81	0.028	(0.017, 0.038)
Senegal	2007-2011	Postneonatal	Niakhar	109	6944	0.014	(0.012, 0.017)	DHS	2017	Fatick	8	966	0.008	(0.002, 0.013)
Senegal	2007-2011	Child	Niakhar	219	27136	0.031	(0.027, 0.035)	DHS	2017	Fatick	22	3667	0.023	(0.013, 0.034)
Senegal	2012-2016	Neonatal	Niakhar	97	606	0.012	(0.010, 0.015)	DHS	2017	Fatick	29	81	0.027	(0.017, 0.037)
Senegal	2012-2016	Postneonatal	Niakhar	93	7353	0.012	(0.010, 0.014)	DHS	2017	Fatick	5	965	0.004	(0.000, 0.008)

Senegal	2012-2016	Child	Niakhar	181	30859	0.023	(0.020, 0.026)	DHS	2017	Fatick	12	4159	0.012	(0.005, 0.019)
Senegal	2008-2012	Neonatal	Bandafassi	55	190	0.022	(0.017, 0.028)	DHS	2018	Kedougou	4	9	0.029	(0.016, 0.041)
Senegal	2008-2012	Postneonatal	Bandafassi	84	2220	0.034	(0.028, 0.042)	DHS	2018	Kedougou	4	107	0.030	(0.014, 0.046)
Senegal	2008-2012	Child	Bandafassi	179	9017	0.076	(0.066, 0.086)	DHS	2018	Kedougou	6	367	0.065	(0.036, 0.093)
Senegal	2008-2012	Neonatal	Mlomp	20	70	0.022	(0.014, 0.033)	DHS	2018	Ziguinchor	12	20	0.046	(0.006, 0.085)
Senegal	2008-2012	Postneonatal	Mlomp	12	794	0.014	(0.008, 0.024)	DHS	2018	Ziguinchor	5	223	0.020	(0.000, 0.039)
Senegal	2008-2012	Child	Mlomp	9	3317	0.011	(0.006, 0.020)	DHS	2018	Ziguinchor	4	839	0.018	(0.000, 0.039)
Senegal	2008-2012	Neonatal	Niakhar	79	591	0.010	(0.008, 0.013)	DHS	2018	Fatick	6	45	0.010	(0.001, 0.018)
Senegal	2008-2012	Postneonatal	Niakhar	110	7103	0.014	(0.012, 0.017)	DHS	2018	Fatick	7	548	0.012	(0.001, 0.024)
Senegal	2008-2012	Child	Niakhar	214	28195	0.029	(0.026, 0.033)	DHS	2018	Fatick	15	2032	0.026	(0.015, 0.038)
South Africa	1993-1997	Neonatal	Agincourt	52	724	0.005	(0.004, 0.007)	DHS	1998	Mpumalanga	7	25	0.021	(0.007, 0.035)
South Africa	1993-1997	Postneonatal	Agincourt	95	8873	0.010	(0.008, 0.012)	DHS	1998	Mpumalanga	7	295	0.021	(0.009, 0.033)
South Africa	1993-1997	Child	Agincourt	157	41369	0.015	(0.013, 0.018)	DHS	1998	Mpumalanga	3	1264	0.011	(0.003, 0.019)
South Africa	2006-2010	Neonatal	Agincourt	123	805	0.012	(0.010, 0.014)	DHS	2016	Mpumalanga	11	25	0.033	(0.014, 0.052)
South Africa	2006-2010	Postneonatal	Agincourt	318	9706	0.030	(0.027, 0.033)	DHS	2016	Mpumalanga	8	289	0.026	(0.010, 0.041)
South Africa	2006-2010	Child	Agincourt	224	39300	0.022	(0.019, 0.025)	DHS	2016	Mpumalanga	5	1043	0.019	(0.005, 0.033)
South Africa	2011-2015	Neonatal	Agincourt	99	947	0.008	(0.007, 0.010)	DHS	2016	Mpumalanga	12	28	0.032	(0.013, 0.050)
South Africa	2011-2015	Postneonatal	Agincourt	148	11566	0.012	(0.010, 0.014)	DHS	2016	Mpumalanga	7	333	0.020	(0.006, 0.034)
South Africa	2011-2015	Child	Agincourt	153	49721	0.012	(0.010, 0.014)	DHS	2016	Mpumalanga	5	1340	0.015	(0.000, 0.029)
South Africa	2006-2010	Neonatal	AHRI	31	698	0.003	(0.002, 0.005)	DHS	2016	Kwazulu-Natal	19	54	0.027	(0.011, 0.043)
South Africa	2006-2010	Postneonatal	AHRI	265	8817	0.027	(0.024, 0.031)	DHS	2016	Kwazulu-Natal	21	621	0.030	(0.010, 0.050)
South Africa	2006-2010	Child	AHRI	196	38180	0.020	(0.017, 0.023)	DHS	2016	Kwazulu-Natal	3	2324	0.005	(0.000, 0.010)
South Africa	2011-2015	Neonatal	AHRI	16	605	0.002	(0.001, 0.003)	DHS	2016	Kwazulu-Natal	12	57	0.016	(0.005, 0.027)
South Africa	2011-2015	Postneonatal	AHRI	114	7947	0.013	(0.011, 0.016)	DHS	2016	Kwazulu-Natal	7	679	0.010	(0.002, 0.018)
South Africa	2011-2015	Child	AHRI	97	37272	0.011	(0.009, 0.013)	DHS	2016	Kwazulu-Natal	2	2925	0.003	(0.000, 0.007)
South Africa	2006-2010	Neonatal	Dikgale	1	79	0.001	(0.000, 0.007)	DHS	2016	Limpopo	7	29	0.018	(0.005, 0.031)
South Africa	2006-2010	Postneonatal	Dikgale	13	1136	0.010	(0.006, 0.017)	DHS	2016	Limpopo	3	342	0.007	(0.000, 0.015)
South Africa	2006-2010	Child	Dikgale	15	4511	0.012	(0.008, 0.020)	DHS	2016	Limpopo	5	1276	0.016	(0.004, 0.028)
South Africa	2011-2015	Neonatal	Dikgale	4	253	0.001	(0.000, 0.003)	DHS	2016	Limpopo	6	30	0.014	(0.001, 0.027)
South Africa	2011-2015	Postneonatal	Dikgale	13	3115	0.004	(0.002, 0.007)	DHS	2016	Limpopo	3	353	0.009	(0.000, 0.017)

South Africa	2011-2015	Child	Dikgale	33	16363	0.008	(0.006, 0.012)	DHS	2016	Limpopo	2	1505	0.006	(0.000, 0.013)
Tanzania	1994-1998	Neonatal	Magu	45	241	0.014	(0.011, 0.019)	DHS	1999	Mwanza	12	21	0.043	(0.023, 0.062)
Tanzania	1994-1998	Postneonatal	Magu	245	3204	0.069	(0.062, 0.077)	DHS	1999	Mwanza	15	240	0.055	(0.024, 0.085)
Tanzania	1994-1998	Child	Magu	223	13082	0.064	(0.057, 0.072)	DHS	1999	Mwanza	9	899	0.036	(0.021, 0.051)
Tanzania	1999-2003	Neonatal	Ifakara	316	861	0.028	(0.025, 0.031)	DHS	2004	Morogoro	15	17	0.067	(0.016, 0.116)
Tanzania	1999-2003	Postneonatal	Ifakara	571	10213	0.050	(0.047, 0.054)	DHS	2004	Morogoro	6	181	0.031	(0.004, 0.057)
Tanzania	1999-2003	Child	Ifakara	622	40562	0.058	(0.054, 0.062)	DHS	2004	Morogoro	6	740	0.031	(0.000, 0.061)
Tanzania	1994-1998	Neonatal	Magu	45	241	0.014	(0.011, 0.019)	DHS	2004	Mwanza	13	37	0.028	(0.014, 0.042)
Tanzania	1994-1998	Postneonatal	Magu	245	3204	0.069	(0.062, 0.077)	DHS	2004	Mwanza	22	406	0.049	(0.014, 0.083)
Tanzania	1994-1998	Child	Magu	223	13082	0.064	(0.057, 0.072)	DHS	2004	Mwanza	27	1474	0.066	(0.043, 0.090)
Tanzania	1999-2003	Neonatal	Magu	67	324	0.016	(0.012, 0.020)	DHS	2004	Mwanza	25	44	0.043	(0.022, 0.063)
Tanzania	1999-2003	Postneonatal	Magu	294	4080	0.065	(0.058, 0.072)	DHS	2004	Mwanza	23	504	0.041	(0.024, 0.057)
Tanzania	1999-2003	Child	Magu	218	16629	0.049	(0.044, 0.056)	DHS	2004	Mwanza	15	1840	0.031	(0.005, 0.056)
Tanzania	1999-2003	Neonatal	Rufiji	381	1035	0.028	(0.025, 0.031)	DHS	2004	Pwani	2	10	0.015	(0.000, 0.033)
Tanzania	1999-2003	Postneonatal	Rufiji	712	11851	0.054	(0.050, 0.057)	DHS	2004	Pwani	3	107	0.029	(0.012, 0.045)
Tanzania	1999-2003	Child	Rufiji	536	42604	0.048	(0.044, 0.052)	DHS	2004	Pwani	4	390	0.039	(0.009, 0.068)
Tanzania	2000-2004	Neonatal	Ifakara	365	935	0.029	(0.027, 0.032)	DHS	2010	Morogoro	8	19	0.030	(0.010, 0.050)
Tanzania	2000-2004	Postneonatal	Ifakara	561	11093	0.046	(0.042, 0.049)	DHS	2010	Morogoro	11	216	0.047	(0.017, 0.077)
Tanzania	2000-2004	Child	Ifakara	643	43233	0.057	(0.053, 0.061)	DHS	2010	Morogoro	5	785	0.022	(0.000, 0.045)
Tanzania	2005-2009	Neonatal	Ifakara	486	1155	0.032	(0.029, 0.035)	DHS	2010	Morogoro	11	19	0.046	(0.020, 0.072)
Tanzania	2005-2009	Postneonatal	Ifakara	547	13800	0.036	(0.033, 0.039)	DHS	2010	Morogoro	7	224	0.027	(0.000, 0.054)
Tanzania	2005-2009	Child	Ifakara	550	56579	0.038	(0.035, 0.041)	DHS	2010	Morogoro	4	926	0.015	(0.000, 0.034)
Tanzania	2000-2004	Neonatal	Magu	75	331	0.017	(0.014, 0.021)	DHS	2010	Mwanza	6	35	0.013	(0.000, 0.025)
Tanzania	2000-2004	Postneonatal	Magu	310	4197	0.066	(0.060, 0.073)	DHS	2010	Mwanza	20	388	0.047	(0.023, 0.071)
Tanzania	2000-2004	Child	Magu	222	17142	0.049	(0.044, 0.056)	DHS	2010	Mwanza	28	1303	0.077	(0.048, 0.105)
Tanzania	2005-2009	Neonatal	Magu	13	329	0.003	(0.002, 0.005)	DHS	2010	Mwanza	10	43	0.017	(0.003, 0.031)
Tanzania	2005-2009	Postneonatal	Magu	168	4198	0.037	(0.032, 0.042)	DHS	2010	Mwanza	17	507	0.030	(0.014, 0.046)
Tanzania	2005-2009	Child	Magu	201	18952	0.041	(0.036, 0.047)	DHS	2010	Mwanza	12	1866	0.026	(0.010, 0.042)
Tanzania	2000-2004	Neonatal	Rufiji	348	1057	0.025	(0.023, 0.028)	DHS	2010	Pwani	1	9	0.009	(0.000, 0.020)
Tanzania	2000-2004	Postneonatal	Rufiji	621	12259	0.046	(0.042, 0.049)	DHS	2010	Pwani	7	116	0.057	(0.030, 0.084)

Tanzania	2000-2004	Child	Rufiji	571	44767	0.048	(0.045, 0.052)	DHS	2010	Pwani	5	386	0.053	(0.025, 0.080)
Tanzania	2005-2009	Neonatal	Rufiji	285	1082	0.020	(0.018, 0.022)	DHS	2010	Pwani	4	11	0.030	(0.008, 0.051)
Tanzania	2005-2009	Postneonatal	Rufiji	400	12755	0.029	(0.026, 0.031)	DHS	2010	Pwani	2	124	0.015	(0.000, 0.032)
Tanzania	2005-2009	Child	Rufiji	574	49059	0.044	(0.041, 0.048)	DHS	2010	Pwani	2	516	0.015	(0.001, 0.030)
Tanzania	2005-2009	Neonatal	Ifakara	486	1155	0.032	(0.029, 0.035)	DHS	2015	Morogoro	1	20	0.004	(0.000, 0.011)
Tanzania	2005-2009	Postneonatal	Ifakara	547	13800	0.036	(0.033, 0.039)	DHS	2015	Morogoro	10	237	0.038	(0.010, 0.064)
Tanzania	2005-2009	Child	Ifakara	550	56579	0.038	(0.035, 0.041)	DHS	2015	Morogoro	8	895	0.031	(0.004, 0.057)
Tanzania	2010-2014	Neonatal	Ifakara	527	1369	0.029	(0.027, 0.032)	DHS	2015	Morogoro	5	21	0.016	(0.000, 0.033)
Tanzania	2010-2014	Postneonatal	Ifakara	420	16661	0.023	(0.021, 0.025)	DHS	2015	Morogoro	7	263	0.025	(0.000, 0.049)
Tanzania	2010-2014	Child	Ifakara	502	76950	0.026	(0.024, 0.028)	DHS	2015	Morogoro	7	1038	0.024	(0.008, 0.041)
Tanzania	2005-2009	Neonatal	Magu	13	329	0.003	(0.002, 0.005)	DHS	2015	Mwanza	17	30	0.043	(0.007, 0.078)
Tanzania	2005-2009	Postneonatal	Magu	168	4198	0.037	(0.032, 0.042)	DHS	2015	Mwanza	7	349	0.017	(0.002, 0.032)
Tanzania	2005-2009	Child	Magu	201	18952	0.041	(0.036, 0.047)	DHS	2015	Mwanza	19	1191	0.058	(0.025, 0.090)
Tanzania	2005-2009	Neonatal	Rufiji	285	1082	0.020	(0.018, 0.022)	DHS	2015	Pwani	5	8	0.046	(0.012, 0.078)
Tanzania	2005-2009	Postneonatal	Rufiji	400	12755	0.029	(0.026, 0.031)	DHS	2015	Pwani	2	101	0.018	(0.001, 0.035)
Tanzania	2005-2009	Child	Rufiji	574	49059	0.044	(0.041, 0.048)	DHS	2015	Pwani	5	383	0.053	(0.011, 0.093)
Tanzania	2010-2014	Neonatal	Rufiji	223	977	0.017	(0.015, 0.020)	DHS	2015	Pwani	4	10	0.028	(0.006, 0.049)
Tanzania	2010-2014	Postneonatal	Rufiji	242	11948	0.019	(0.016, 0.021)	DHS	2015	Pwani	3	113	0.022	(0.002, 0.041)
Tanzania	2010-2014	Child	Rufiji	353	53507	0.026	(0.024, 0.029)	DHS	2015	Pwani	2	440	0.015	(0.000, 0.032)
Uganda	2006-2010	Neonatal	Iganga Mayuge	238	739	0.024	(0.022, 0.028)	DHS	2011	Busoga	26	74	0.027	(0.015, 0.038)
Uganda	2006-2010	Postneonatal	Iganga Mayuge	380	9116	0.038	(0.034, 0.041)	DHS	2011	Busoga	14	862	0.015	(0.007, 0.024)
Uganda	2006-2010	Child	Iganga Mayuge	546	39247	0.054	(0.050, 0.058)	DHS	2011	Busoga	30	3330	0.035	(0.022, 0.049)
Uganda	2006-2010	Neonatal	Iganga Mayuge	238	739	0.024	(0.022, 0.028)	DHS	2016	Busoga	39	99	0.029	(0.015, 0.044)
Uganda	2006-2010	Postneonatal	Iganga Mayuge	380	9116	0.038	(0.034, 0.041)	DHS	2016	Busoga	33	1180	0.025	(0.017, 0.034)
Uganda	2006-2010	Child	Iganga Mayuge	546	39247	0.054	(0.050, 0.058)	DHS	2016	Busoga	35	4559	0.030	(0.019, 0.040)
Uganda	2011-2015	Neonatal	Iganga Mayuge	294	786	0.028	(0.025, 0.032)	DHS	2016	Busoga	39	107	0.028	(0.020, 0.036)
Uganda	2011-2015	Postneonatal	Iganga Mayuge	288	10000	0.026	(0.023, 0.029)	DHS	2016	Busoga	34	1248	0.025	(0.013, 0.036)
Uganda	2011-2015	Child	Iganga Mayuge	424	45835	0.037	(0.033, 0.040)	DHS	2016	Busoga	41	5117	0.031	(0.021, 0.042)

Note -- PY: person-years, CI: 95% confidence intervals calculated using Taylor linearisation.
